# Shared and more specific genetic determinants and pathways underlying yeast tolerance to acetic, butyric, and octanoic acids

**DOI:** 10.1186/s12934-024-02309-0

**Published:** 2024-02-29

**Authors:** Marta N. Mota, Madalena Matos, Nada Bahri, Isabel Sá-Correia

**Affiliations:** 1 iBB—Institute for Bioengineering and Biosciences, Instituto Superior Técnico, Universidade de Lisboa, Av. Rovisco Pais, 1, 1049-001 Lisbon, Portugal; 2 Department of Bioengineering, Instituto Superior Técnico, Universidade de Lisboa, Av. Rovisco Pais, 1, 1049-001 Lisbon, Portugal; 3 i4HB—Institute for Health and Bioeconomy, Instituto Superior Técnico, Universidade de Lisboa, Av. Rovisco Pais, 1, 1049-001 Lisbon, Portugal

**Keywords:** Chemogenomic analysis, Toxicity mechanisms, Weak acids, Volatile fatty acids, Yeast tolerance, Genome engineering, Superior yeasts

## Abstract

**Background:**

The improvement of yeast tolerance to acetic, butyric, and octanoic acids is an important step for the implementation of economically and technologically sustainable bioprocesses for the bioconversion of renewable biomass resources and wastes. To guide genome engineering of promising yeast cell factories toward highly robust superior strains, it is instrumental to identify molecular targets and understand the mechanisms underlying tolerance to those monocarboxylic fatty acids. A chemogenomic analysis was performed, complemented with physiological studies, to unveil genetic tolerance determinants in the model yeast and cell factory *Saccharomyces cerevisiae* exposed to equivalent moderate inhibitory concentrations of acetic, butyric, or octanoic acids.

**Results:**

Results indicate the existence of multiple shared genetic determinants and pathways underlying tolerance to these short- and medium-chain fatty acids, such as vacuolar acidification, intracellular trafficking, autophagy, and protein synthesis. The number of tolerance genes identified increased with the linear chain length and the datasets for butyric and octanoic acids include the highest number of genes in common suggesting the existence of more similar toxicity and tolerance mechanisms. Results of this analysis, at the systems level, point to a more marked deleterious effect of an equivalent inhibitory concentration of the more lipophilic octanoic acid, followed by butyric acid, on the cell envelope and on cellular membranes function and lipid remodeling. The importance of mitochondrial genome maintenance and functional mitochondria to obtain ATP for energy-dependent detoxification processes also emerged from this chemogenomic analysis, especially for octanoic acid.

**Conclusions:**

This study provides new biological knowledge of interest to gain further mechanistic insights into toxicity and tolerance to linear-chain monocarboxylic acids of increasing liposolubility and reports the first lists of tolerance genes, at the genome scale, for butyric and octanoic acids. These genes and biological functions are potential targets for synthetic biology approaches applied to promising yeast cell factories, toward more robust superior strains, a highly desirable phenotype to increase the economic viability of bioprocesses based on mixtures of volatiles/medium-chain fatty acids derived from low-cost biodegradable substrates or lignocellulose hydrolysates.

**Supplementary Information:**

The online version contains supplementary material available at 10.1186/s12934-024-02309-0.

## Introduction

The improvement of the tolerance of industrially relevant yeast strains to multiple stresses imposed by toxic feedstocks and (by)products is a major challenge of modern Biotechnology [1–3]. In this context, scientific research and innovation, at the biological level, are required to understand the genetic determinants and signaling pathways underlying yeast robustness under weak acid stress. This type of stress is highly relevant for several reasons, depending on the specific compound. Acetic acid (C2) accumulates in hydrolysates from diverse biomasses with acetylated sugar backbone chains being considered a main inhibitor in 2nd generation (2G) biorefinery processes [4–6]. Acetic acid can also be used as a carbon (C) source by several yeast species [7, 8], is an inhibitory product of yeast metabolism [9] and is widely used as food preservative [10]. Formic acid (C1) is another short-chain inhibitory weak acid present in lignocellulosic hydrolysates [6]. Several chemogenomic and genomic expression remodeling analyses identified genetic and physiological determinants of yeast tolerance to acetic acid stress [11–13] and also to formic acid [14–16]. Results indicate that there are multiple shared determinants and pathways underlying tolerance to these two short-chain weak acids but there are also more specific genetic determinants [12, 16]. Other volatile fatty acids (VFAs) (linear short-chain aliphatic mono-carboxylate compounds, having from one to seven carbon atoms), in particular butyric acid (C4), are intermediary metabolites generated through anaerobic digestion of a wide variety of organic wastes, being promising building blocks of zero or even negative cost and valuable compounds of the biofuel industry [17, 18]. The medium-chained fatty acid (MCFA) octanoic acid (C8) is a fermentation inhibitor that can lead to stuck wine fermentation [19, 20], and a metabolite of *S. cerevisiae* metabolism of high economic interest [21, 22]. Short-and medium- chain fatty acids can be used as a platform to generate petroleum-derived building blocks [23–25]. The toxicity of short- to medium- linear chain fatty acids correlates with their lipophilic properties, which indicates a deleterious effect on cell membrane organization and function [19, 23]. Depending on the linear monocarboxylic acid carbon chain length, there are specific toxicity and tolerance genetic determinants involved in stress response [19]. The penetration of these acids inside the cell in the nonionized form by passive diffusion is followed by their dissociation in the cytosol, at a pH quite above the weak acid pKa, leading to intracellular acidification and the accumulation of the acid counterion [12, 26]. Energy-dependent mechanisms are induced to counteract these and other toxicity effects [12, 26, 27]. Robust yeast strains leading to high product yields and titers are desirable to increase bioprocesses´ economic viability [23, 24]. For this reason, it is fundamental to attain a better understanding of the molecular targets and pathways behind increased yeast tolerance to guide the rational increase of yeast robustness in the presence of these fatty acids by genome engineering. In particular, this is essential knowledge to improve the competitiveness of bioprocesses based on lignocellulosic biomass hydrolysates or VFA/MCFA mixtures derived from organic wastes [1, 3].

*S. cerevisiae* is an essential experimental model yeast widely employed in biotechnology. However, natural strains lack many desired traits to be useful for a circular bioeconomy, in particular, the capacity to use a wide range of C-sources, special metabolic potential, and high multi-stress tolerance [28–30]. Therefore, the interest in non-*Saccharomyces* yeasts is gaining momentum whenever they exhibit those features [28–30]. Nevertheless, the model yeast continues essential to facilitate the mechanistic understanding of toxicity and tolerance mechanisms in yeasts. The molecular mechanisms underlying *Saccharomyces cerevisiae* response and adaptation to acetic acid have been studied for years. However, only recently these studies were extended to non-*Saccharomyces* yeasts such as the remarkably weak acid-tolerant species, *Zygosaccharomyces bailii* and *Zygosaccharomyces parabaillii*, responsible for food and beverage spoilage [29–32]. A holistic view on the responses and tolerance mechanisms to acetic acid in those yeast species, compared with *S. cerevisiae,* is available [12].The aim of present research work was to contribute to the understanding of weak acids toxicity and tolerance in the model yeast and cell factory, *S. cerevisiae,* by comparing the underlying genetic determinants in short (C4-butyric acid) and medium (C8-octanoic acid) straight-linear chain monocarboxylic weak acids, compared with acetic acid. A chemogenomic analysis was performed to identify shared and more specific molecular determinants and signalling pathways underlying *S. cerevisiae* tolerance to equivalent levels of stress imposed by these acids. The Euroscarf yeast deletion collection tested was already screened for susceptibility to acetic acid stress by our laboratory and others [11, 33, 34]. However, differences in the level of stress and experimental approach may lead to different, even conflicting, results, as it is for example the case of two chemogenomic analyses performed to identify acetic acid tolerance genes using moderate sub-lethal stress conditions [11] or lethal conditions [33]. Considering the objective of this study and to address this issue, equivalent inhibitory concentrations of the three acids and the same experimental conditions were used to obtain the corresponding genetic determinants of tolerance. This study is expected to provide further global mechanistic insights into their toxic effects and on how the yeast cell express its tolerance towards their deleterious action. Moreover, the genes and biological functions emerging from the exploration of this genome-wide approach, in particular those corresponding to butyric and octanoic acid here reported for the first time, are potential targets for reverse genetic engineering of promising yeast cell factories, toward highly robust superior strains, able to withstand harsher conditions. The candidate molecular targets identified in the yeast model can also be explored for rational genetic manipulations of non-conventional yeasts aiming at the improvement of performance in relevant biotechnological and food industry processes.

## Results

### Time-course effects of acetic, butyric and octanoic acids in yeast physiology during growth

**Fig. 1:**
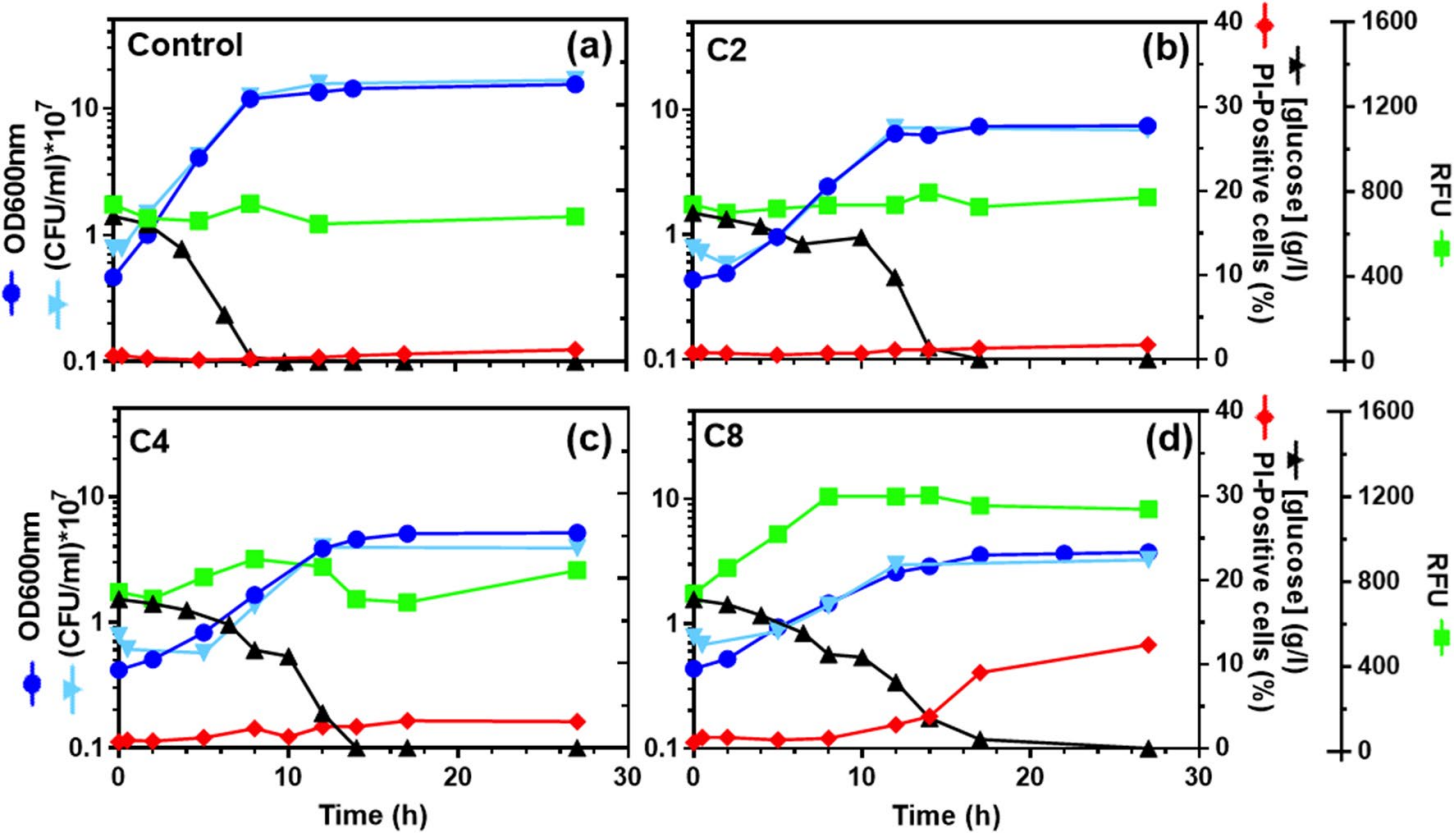
Time-course effect of acetic, butyric and octanoic acids in yeast physiology during the growth curve. The progress of *S. cerevisiae* BY4741pHl growth in YPD (pH 4.5) in the absence (**a**) or presence of 62 mM (3.78 g/L) acetic acid (**b**), 15.04 mM (1.33 g/L) butyric acid (**c**) or 0.47 mM (0.07 g/L) octanoic acid (**d**), at 30 ℃, with orbital agitation, were followed based on culture OD_600nm_ (dark blue circles), CFU/mL (light blue triangles), and glucose consumption (black triangles). The variation of cell permeability (RFU, green squares) and the percentage of PI-Positive cells (red diamonds) during cultivation are also shown. Results are representative of at least three independent experiments carried out

Following previous growth experiments to examine the dose–effect of acetic acid, butyric acid, or octanoic acid in the yeast growth curve, the impact in growth and physiology of selected equivalent concentrations of these acids was assessed. For this, *S. cerevisiae* BY4741pHl was cultivated in YPD medium at pH 4.5 **(**Fig. 1a) or in this medium supplemented with 62 mM (3.78 g/L) acetic acid (Fig. 1b), 15.04 mM (1.33 g/L) butyric acid (Fig. 1c), or 0.47 mM (0.07 g/L) octanoic acid (Fig. 1d). The marked differences in the equivalent weak acid concentrations used are consistent with the well-known increased toxicity of these monocarboxylic acids with the increase of the linear carbon-chain. Cell growth was followed based on culture OD_600nm_, complemented by the concentration of viable cells (as concentration of colony forming units) and glucose consumption during the growth curve. In the presence of the weak acids, the final biomass, and the maximum specific growth rate, based on both the culture OD_600nm_ and the concentration of viable cells, were below the values registered in absence of stress. However, the induced lag phase was barely detectable under the relatively mild inhibitory concentrations tested. The final biomass attained by the three cultures when the carbon source, glucose, was exhausted, was below the attained in the absence of stress. This effect was more evident  under octanoic acid stress, but was also found for butyric acid and acetic acid, although the effect was less marked, especially for acetic acid (Fig. 1), indicating a higher energy dissipation in stress-responsive mechanisms whenan equivalent level of stress is induced by longer chain fatty acids.

Although the objective was the use of equivalent inhibitory concentrations of the three acids, it was not possible to obtain identical growth curves. During the first hours of cultivation, it was possible to obtain similar growth curves but, as the cultivation under stress progressed, a more marked inhibitory effect was observed for butyric acid and especially for octanoic acid (Fig. 1). The higher liposolubility of these carboxylic acids as the C-chain increases [37] implicated the use of much lower equivalent concentrations of butyric acid and, especially, of octanoic acid in the growth medium. The results indicating an increased toxicity of the more lipophilic acids as growth proceeds, suggest an increased accumulation of the low-concentration more lipophilic weak acids at the plasma membrane [35] over cultivation in their presence. This hypothesis is consistent with the observed significant increase of plasma membrane permeability, analyzed by flow cytometry following staining with propidium iodide (PI), over the first part of exponential growth especially for octanoic acid stressed cells (Fig. 1). Although not so marked, such an increase of permeability was also detected for butyric acid stressed cells during exponential growth but not under acetic acid stress. The percentage of PI-positive cells (the percentage of M2 sub-population of highly permeabilized cells, as described in the Methods section) was also found to increase during exponential growth under octanoic acid stress, followed by the action of butyric acid stress. Remarkably, no detectable decrease in cell viability, assessed by the concentration of colony forming units, was found for butyric acid or even for octanoic acid stressed cultures during exponential growth, suggesting that those highly permeable cells were still able to recover and duplicate in inhibitor-free rich medium [35].

**Fig. 2:**
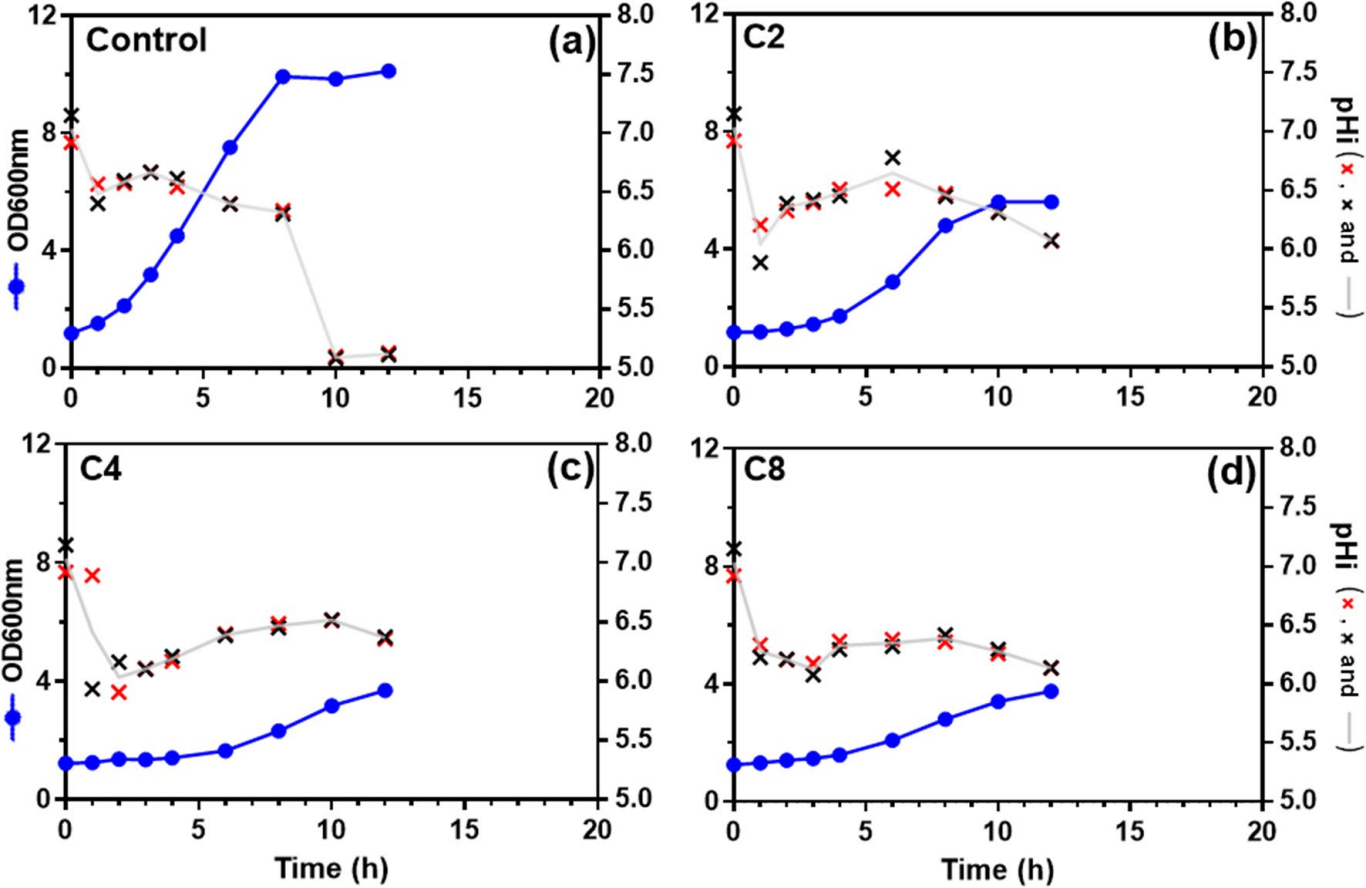
Time-course effect of acetic, butyric and octanoic Acids in yeast intracellular pH. Growth curve and intracellular pH (pHi, black and red crosses, and grey line) variation during *S. cerevisiae* BY4741pHl growth in MM (pH 4.5) in the absence (**a**) or presence of 53 mM (3.23 g/L) acetic acid (**b**), 11 mM (0.97 g/L) butyric acid (**c**) or 0.43 mM (0.06 g/L) octanoic acid (**d**) at 30 ℃ with orbital agitation. Cell growth was based on OD_600nm_ (dark blue circles). Results from two representative experiments performed to obtain the pHi profiles are shown as the values of each replicate (black and red crosses) and as the calculated average of the two values (grey line)

The effect exerted by the three weak acids in the intracellular pH (pHi) during cultivation was also compared (Fig. 2) by determining the I405/I465 ratio of yeast cell cultures growing in a medium adequate to fluorescence measurements (MM medium), supplemented with adjusted equivalent concentrations of the three acids, at pH 4.5. A steep decrease of pHi was observed one hour after the beginning of cultivation, this decrease having a higher amplitude under weak acid stress conditions (from 7 to around 6), as recently reported for cultivation under acetic acid stress [36]. The beginning of sustainable exponential growth was accompanied by an increase in pHi up to about 6.5. When entering the stationary phase a decrease in pHi was observed, which is more visible in the control conditions when cell biomass reach maximal values (Fig. 2a), as described in other studies [36, 37]. The recovery of pHi from 6 to 6.5 appears to be slower for cells growing in presence of butyric acid or octanoic acid. All together, these results indicate that, independently of the slight differences observed, internal acidification does not appear to be a major distinctive effect for cells adapted to the short and medium-chain fatty acids tested. Moreover, for the moderate level of the weak acid stress examined, pHi does not appear to be a major underlying deleterious mechanism since the yeast cells were able to counteract intracellular acidification.

### Selection of equivalent concentrations of acetic, butyric and octanoic acids for the chemogenomic analysis

**Fig. 3:**
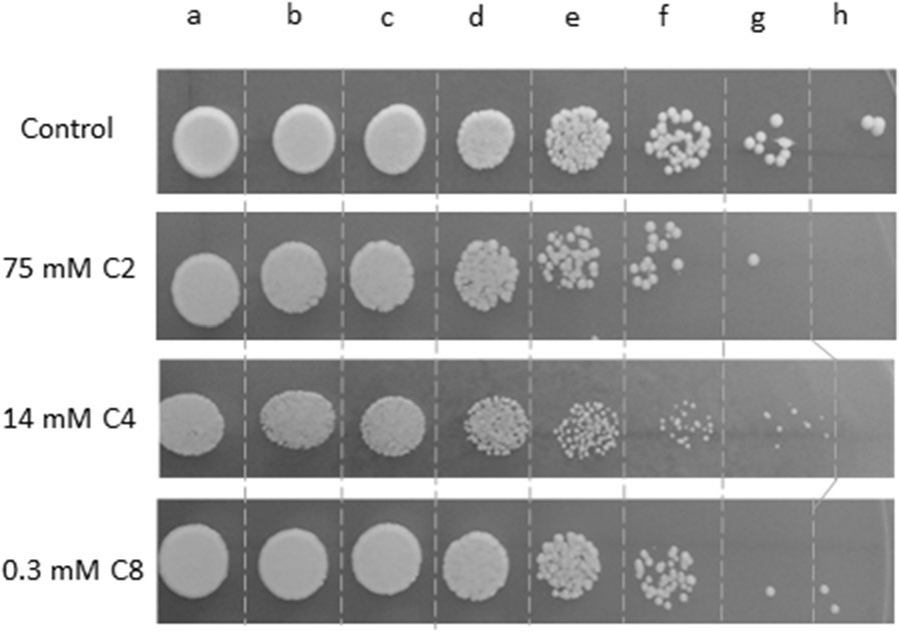
Equivalent growth inhibitory concentrations of the weak acids used for the chemogenomic analysis. Spot growth of *S. cerevisiae* BY4741 cultivated in YPD solid medium (pH 4.5) either or not supplemented with acetic acid (75 mM C2), butyric acid (14 mM C4), or octanoic acid (0.3 mM C8). Exponentially-growing cell suspensions of BY4741 (OD_600nm_ of 1 ± 0.05) were diluted to an OD_600nm_ of 0.5 ± 0.005 (**a**) and this suspension was used to prepare 1:2 (**b**), 1:4 (**c**), 1:20 (d), 1:100 (**e**), 1:500 (**f**), 1:2500 (**g**) and 1:12,500 (**h**) diluted suspensions. Spot growth was registered after 48 h of incubation at 30 ℃. This picture is a representative example of several independent growth experiments

The appropriate equivalent concentrations of acetic, butyric, and octanoic acids to perform the chemogenomic analysis were selected by testing the parental strain *S. cerevisiae* BY4741 by spot assays, as described in the Methods section. Results from testing the parental yeast strain susceptibility to increasing concentrations of the weak acids are shown in Additional file 16: Figure S1. Equivalent mild growth inhibitory concentrations for the parental strain were found by the supplementation of YPD solid medium at pH 4.5 with 75 mM (4.58 g/L) of acetic acid, or 14 mM (1.23 g/L) of butyric acid, or 0.3 mM (0.04 g/L) of octanoic acid (Fig. 3). These acid concentrations were used for the planned genome-wide analysis.

### Identification, at genome-wide scale, of genes required for maximum tolerance to acetic, butyric or octanoic acids

Approximately 5100 *S.cerevisiae* BY4741 single deletion mutants of the Euroscarf haploid deletion mutant collection were tested for susceptibility to the above referred monocarboxylic acid concentrations, compared with the parental strain. Concerning acetic acid stress, 377 deletion mutants were found to be more susceptible than the parental strain, 46 of them showing total growth inhibition (+ +), and 331 a minor to moderate growth inhibition ( +). In presence of butyric acid, 422 deletion mutants were found to be more susceptible with 51 deletion mutants exhibiting full growth inhibition and 371 a minor to moderate susceptibility phenotype. For octanoic acid, 490 deletion mutants were found to be more susceptible, with 53 of those leading to total growth inhibition and 437 showing a minor to moderate susceptibility phenotype. A visual description of the criteria used to define the different levels of susceptibility to acetic (C2), butyric (C4) and/or octanoic (C8) acids of the different deletion mutant strains tested is shown in Additional file 2: Figure S2.The complete lists of genes required for maximum tolerance to these monocarboxylic acids are available in the Additional file 1: Table S1, Additional file 2: S2 and Additional file 3: S3, respectively. No gene that when deleted led to higher weak acid tolerance was detected.

**Fig. 4:**
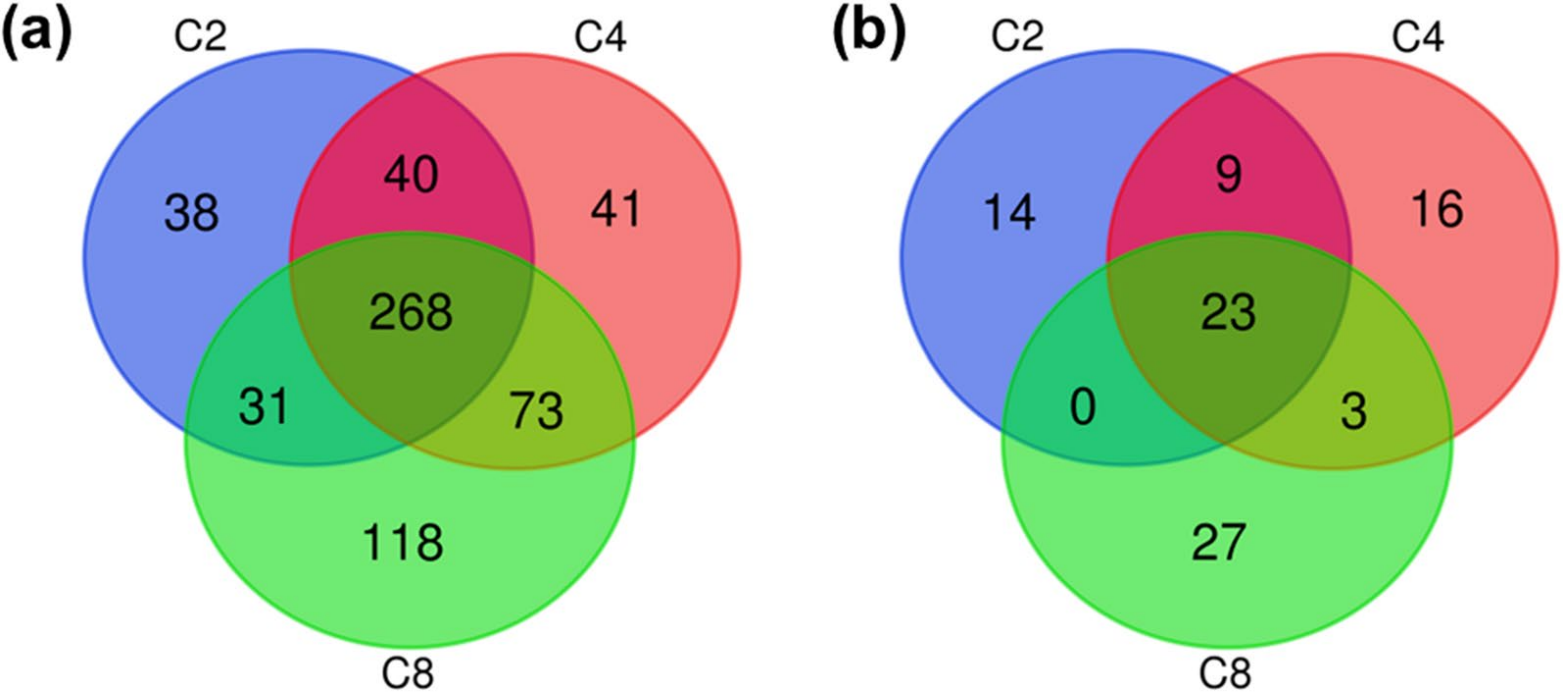
Diagram representing the number of specific or shared tolerance genes. The chemogenomic analysis was performed for equivalent concentrations of acetic acid (C2) (blue), butyric acid (C4) (red), and octanoic acid (C8) (green). Panel (**a)** refers to all the tolerance genes obtained for the three weak acids while panel (**b)** refers to the genes whose deletion led to the +  + phenotype

The comparison of the obtained datasets allowed the single out of genes that confer tolerance to the three weak acids and of those that are specific to each dataset or to a combination of two datasets (Fig. 4a). The complete lists of genes shared between datasets and genes exclusive of one dataset are available in the Additional file 4: Table S4, Additional file 5: Table S5, Additional file 6: Table S6, Additional file 7: Table S7, Additional file 8: Table S8, Additional file 9: Table S9, Additional file 10: Table S10). A significant overlap of the tolerance determinants was observed for the three acids (268 genes). In addition to the genes shared by the three datasets, the butyric acid and octanoic acid datasets shared the highest number of identified genes in common (71) compared with any other combination of two acids. This suggests a higher similarity in toxicity and tolerance mechanisms. It was also found that the octanoic acid dataset exhibits the highest number of genes whose expression is specifically required for maximum tolerance to octanoic acid (118), compared with butyric acid [41] and acetic acid [38]. The general conclusions that can be taken from the Ven diagram representing only the genes whose deletion led to full growth inhibition (Fig. 4b) are, in general, similar to those taken from the diagram that includes all the tolerance genes (Fig. 4a).

**Fig. 5:**
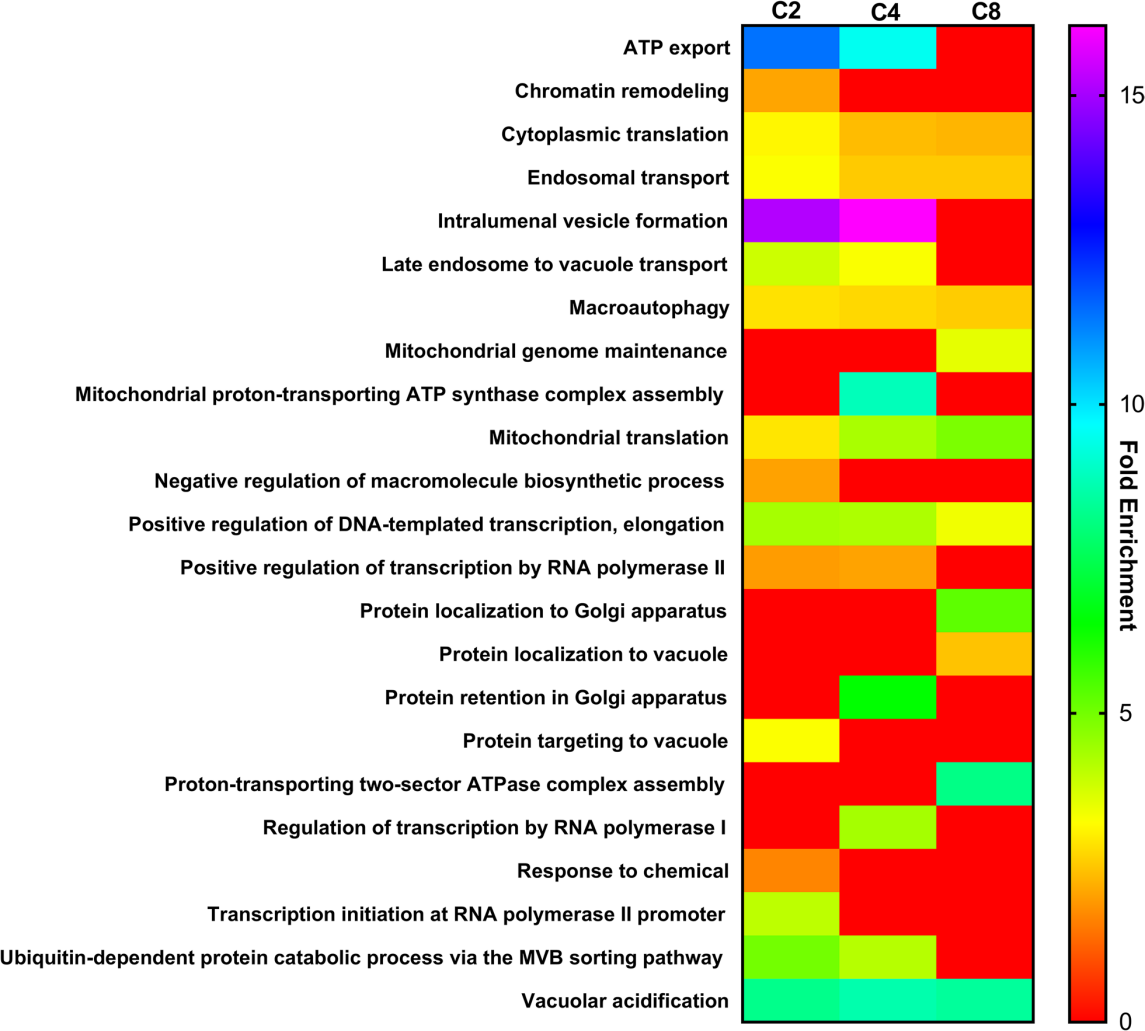
Biological functions enriched in the datasets obtained from the chemogenomic analysis performed. The datasets include genes found to be required for maximum yeast tolerance to 75 mM acetic acid (C2), 14 mM butyric acid (C4), or 0.3 mM octanoic acid (C8) at pH 4.5. Genes listed in, Additional file 1: Table S1, Additional file 2: Table S2, and Additional file 3: Table S3, were clustered according to the corresponding biological process GO assignments using the PANTHER Classification System (http://pantherdb.org), and functional categories were considered to be over-represented if the p-value < 0.05. The fold enrichment is calculated by dividing the number of genes present in the input dataset by the total number of genes of the yeast genome expected to belong to a specific functional class

Based on the biological function of the genes identified as required for maximum acetic acid, butyric acid or octanoic acid tolerance, they were clustered according to the PANTHER Classification System [38]. The fold enrichment of different functional classes in the three datasets (p-value < 0.05), is shown in Fig. 5. It was also tried to perform the same functional analysis but only dedicated to those genes whose deletion led to a +  + phenotype to try to obtain meaningful insights. However, the analysis of this smaller group of genes did not provide significant results according to the PANTHER Classification System, except for the octanoic acid dataset for which one single function was found to be significantly enriched. The corresponding function “Organic substance biosynthetic process” includes 27 genes and the functions of some of them (e.g. *ERG2, ERG28*, *CSG2, SUR1 ARG82 ELO2, OCH1, MNN9, MNN10*) point to the relevance of cell membranes, cell lipids and cell wall as major tolerance determinants more specific for the more lipophilic weak acid tested.

A more detailed discussion on selected tolerance genes, in particular those belonging to differently enriched functional classes in Fig. 5, follows.

### Genes involved in vacuolar and vesicular function and transport

The functional analysis revealed ten functions related to vacuolar and vesicular transport required for maximum tolerance to the tested weak acids. These functions include “ATP export”, “Ubiquitin-dependent protein catabolic process via the Multivesicular Body (MVB) sorting pathway”, “Intraluminal vesicle formation”, “Late endosome to vacuole transport”, “Endosomal transport”, “Protein localization to Golgi apparatus”, “Protein localization to vacuole”, “Protein targeting to vacuole”, “Protein retention in Golgi apparatus”. They include a core of 52 genes, involved in one or more of these functions (Additional file 11: Table S11). At least, two of these functions are shared by 33 genes while 17 genes are shared by at least three of those functions (are shown in Table 1). The functions “Endosomal transport” and “Vacuolar acidification” are enriched in the three datasets **(**Fig. 5**)**. Seventeen genes are included in “Vacuolar acidification" function, most of them encoding subunits of the multimeric vacuolar H^+^-ATPase or required for its assembly (*DBF2*, *MEH1*, *PFK2*, *RAV2*, *RRG1*, *VMA1*, *VMA10*, *VMA11*, *VMA13*, *VMA16*, *VMA2*, *VMA3*, *VMA5*, *VMA7*, *VPH1*, *VPH2*, *VPS3*) **(**Table 2**)**. The functions “ATP export” and “Intralumenal vesicle formation” displayed the highest level of fold enrichment and, along with “Ubiquitin-dependent protein catabolic process via the MVB sorting pathway” and “Late endosome to vacuole transport”, were identified in both acetic acid and butyric acid datasets with no significant enrichment in the octanoic acid dataset (Fig. 5). The function “Protein targeting to vacuole” was only significantly enriched in the acetic acid dataset and the function “Protein retention in Golgi apparatus” is exclusive of the butyric acid dataset. The functions “Protein localization to Golgi apparatus” and “Protein localization to vacuole” were exclusively found in the octanoic acid dataset.

**Table 1:** Genes involved in vacuolar and vesicular function and transport

Gene/ORF	Description of the encoded protein function	Susceptibility		
		C2	C4	C8
*ARL1 *(5,6,7)	Soluble GTPase with a role in regulation of membrane traffic. Arl1p regulates potassium influx and also plays a role in membrane organization at trans-Golgi network	+	0	+
*ATG20* (4,6,7)	Sorting nexin family member. Atg20p is required for the cytoplasm-to-vacuole targeting (Cvt) pathway, endosomal sorting and selective autophagy	+	+	+
*BRO1* (1,2,3,4,6)	Cytoplasmic class E vacuolar protein sorting (VPS) factor. Bro1p coordinates protein sorting and deubiquitination in the multivesicular body (MVB) pathway by recruiting Doa4p to endosomes	+	+	+
*DID4* (1,2,3,4,8)	Class E Vps protein of the ESCRT-III complex. Did4p is required for sorting of integral membrane proteins into lumenal vesicles of multivesicular bodies, and for delivery of newly synthesized vacuolar enzymes to the vacuole. Did4p is also involved in endocytosis	+	+	0
*SNF7* (1,2,3,4)	One of four subunits of the ESCRT-III complex. Snf7p is involved in the sorting of transmembrane proteins into the multivesicular body (MVB) pathway. Snf7p is recruited from the cytoplasm to endosomal membranes	+	+	+
*SNF8* (1,3,4,7)	Component of the ESCRT-II complex. Snf8p is involved in glucose derepression	+	+	0
*STP22* (1,3,6,7)	Component of the ESCRT-I complex	+	+	+ +
*VPS1 *(5,6,7,8)	Dynamin-like GTPase required for vacuolar sorting. Vps1p promotes fission of retrograde transport carriers from endosome. It is also involved in endocytosis and late Golgi-retention of some proteins	+	+	+
*VPS16* (3,4,6,7)	Subunit of the HOPS and the CORVET complexes and also part of the Class C Vps complex essential for membrane docking and fusion at Golgi-to-endosome and endosome-to-vacuole protein transport stages	+	+ +	+ +
*VPS20* (1,2,3,4)	Myristoylated subunit of the ESCRT-III complex. Vps20p is a cytoplasmic protein recruited to endosomal membrane	+	+	+
*VPS24* (1,2,3,4)	One of four subunits of the ESCRT-III complex. Vps24p forms an endosomal sorting complex required for transport III (ESCRT-III) subcomplex with Did4p. Vps24p is also involved in the sorting of transmembrane proteins into the multivesicular body (MVB) pathway	+	+	−
*VPS25* (1,3,4,7)	Component of the ESCRT-II complex	+	C4	−
*VPS27* (1,3,4,5,6,8)	Ubiquitin binding protein involved in endosomal protein sorting; subunit of the ESCRT-0 complex that binds to ubiquitin. Vps27p is required for recycling Golgi proteins, forming lumenal membranes and sorting ubiquitinated proteins destined for degradation	−	+	+
*VPS28* (1,3,4,7)	Component of the ESCRT-I complex	+	+	−
*VPS36* (1,3,4,7)	Component of the ESCRT-II complex. Vps36p contains the GLUE domain which is involved in interactions with ESCRT-I and ubiquitin-dependent sorting of proteins into the endosome	+	−	−
*VPS4* (2,3,4,5,8)	AAA-ATPase involved in multivesicular body (MVB) protein sorting. Vps4p localizes to endosomes and catalyzes ESCRT-III disassembly and membrane release	−	+ +	+
*YPT6* (4,6,7)	Rab family GTPase, required for endosome-to-Golgi, intra-Golgi retrograde, and retrograde Golgi-to-ER transport	+	+	+

The table includes the list of genes, involved in vacuolar acidification, identified in this study as determinants of yeast tolerance to acetic acid (C2), butyric acid (C4), or octanoic acid (C8). The description of the encoded protein functions is based on the information at SGD. The susceptibility phenotype of each single deletion mutant was scored, after 48 h as described in Additional Files, Figure S2: ( +) if the mutant strain showed, compared with the parental strain, a slight to moderate growth inhibition, and (+ +) if no growth was observed. “0” corresponds to an absence of a detectable susceptibility phenotype. Underneath the name of each gene are indicated the functions, as defined in the PANTHER Classification System (http://pantherdb.org) used for functional analysis, to which the gene is correlated

The table includes the list of genes, involved in vacuolar and vesicular function and transport, identified in this study as determinants of yeast tolerance to acetic acid (C2), butyric acid (C4), or octanoic acid (C8)

(1) “ATP export” and “Ubiquitin-dependent protein catabolic process via the MVB sorting pathway” (C2/C4)

(2) “intraluminal vesicle formation” (C2/C4)

(3) “Late endosome to vacuole transport” (C2/C4)

(4) “Endosomal Transport” (C2/C4/C8)

(5) “Protein localization to Golgi apparatus” (C8)

(6) “Protein localization to vacuole” (C8)

(7) “Protein targeting to vacuole” (C2)

(8) “Protein retention in Golgi apparatus” (C4)

**Table 2:** Genes involved in vacuolar acidification

Gene/ORF	Description of the encoded protein function	Susceptibility		
		C2	C4	C8
*DBF2*	Ser/Thr kinase involved in transcription and stress response; functions as part of a network of genes in exit from mitosis	+	+	+
*MEH1*	Subunit of EGO/GSE complex; vacuolar/endosomal membrane-associated EGO/GSE complex regulates exit from rapamycin-induced growth arrest	0	0	+
*PFK2*	Beta subunit of heterooctameric phosphofructokinase, involved in glycolysis	+ +	+ +	+ +
*RAV2*	Subunit of RAVE complex	0	+	+
*RRG1*	No demonstrated function but Rrg1p may be required for vacuolar acidification, efficient 5′ processing of mitochondrial tRNAs, for respiratory growth and mitochondrial genome maintenance	0	+	+
*VMA1*	Subunit A of the V1 peripheral membrane domain of V-ATPase	+	+	+
*VMA10*	Subunit G of the V1 peripheral membrane domain of V-ATPase. Vma10p is involved in vacuolar acidification	+ +	+	+
*VMA11*	Vacuolar ATPase V0 domain subunit c′. Vma11p is involved in proton transport activity	+	+	+
*VMA13*	Subunit H of the V1 peripheral membrane domain of V-ATPase. Vma13p serves as an activator or a structural stabilizer of the V-ATPase	0	0	+
*VMA16*	Subunit c′′ of the vacuolar ATPase	+	+	+
*VMA2*	Subunit B of V1 peripheral membrane domain of vacuolar H^+^-ATPase	+	+	+
*VMA3*	Proteolipid subunit c of the V0 domain of vacuolar H^+^ -ATPase. Vma3p is required for vacuolar acidification	+	+	+
*VMA5*	Subunit C of the V1 peripheral membrane domain of V-ATPase. Vma5p is required for the V1 domain to assemble onto the vacuolar membrane	+	+	+
*VMA7*	Subunit F of the V1 peripheral membrane domain of V-ATPase. Vma7p is required for the V1 domain to assemble onto the vacuolar membrane;	+	+	+
*VPH1*	Subunit a of the vacuolar-ATPase V0 domain located in vacuolar V-ATPase complexes	+	+	+
*VPH2*	Integral membrane protein required for V-ATPase function. Vph2p is involved in the assembly of the V-ATPase	+	+	+
*VPS3*	Component of CORVET membrane tethering complex. Vps3p is involvedin the acidification of the vacuolar lumen and in the assembly of the vacuolar H^+^-ATPase	+	+	+

The table includes the list of genes, involved in vacuolar acidification, identified in this study as determinants of yeast tolerance to acetic acid (C2), butyric acid (C4), or octanoic acid (C8). Table elaborated as described in Table 1

### Genes involved in macroautophagy

The three datasets are enriched in genes related to “Macroautophagy'' function. In this category, 26 genes were identified and are listed on Additional file 12: Table S12. Macroautophagy is the most prevalent form of autophagy in which the substrates (superfluous and damaged organelles, cytosolic proteins), following degradation, are released back into the cytosol in order to recycle the macromolecular constituents and to generate energy to maintain cell viability under unfavorable conditions, thus protecting the cell under stress [39].

### Genes involved in transcription and transcription regulation

The transcription and transcription regulation classes include six functions gathering a total of 77 genes (Additional file 13: Table S13), 33 of which are common to at least two of the above referred biological functions **(**Table 3**)**. Several enriched functions related to transcription regulation and transcription initiation/elongation were found to be relevant for acetic acid, butyric acid or octanoic acid tolerance. The functions “Transcription initiation at RNA polymerase II promoter” and “Positive regulation of transcription by RNA polymerase II” are enriched in acetic acid and butyric acid datasets, sharing six genes (*SRB5, SIN4, MED2, GAL11, DST1, CSE2*) that encode subunits of the RNA polymerase II mediator complex (except *GAL11*). The functions “Chromatin remodeling” and “Negative regulation of macromolecule biosynthetic process” are exclusively enriched in the acetic acid dataset, while “Regulation of transcription by RNA polymerase I” is exclusively enriched in the butyric acid dataset. RNA polymerase I synthesizes 60% of cellular RNA by transcribing several copies of the rRNA gene and it is a key determinant for the level of ribosome components, controlling cell growth [40]. The “Positive regulation of DNA-templated transcription, elongation” function is enriched in all datasets.

**Table 3:** Genes involved in the transcription process

Gene/ ORF	Description of the encoded protein function	Susceptibility		
		C2	C4	C8
*ASF1* (1,4,6)	Nucleosome assembly factor that is involved in chromatin assembly and disassembly. Asf1p is required for recovery after double strand break repair	+	0	0
*BUD27* (1,5)	Unconventional prefoldin protein involved in translation initiation. Bud27p is required for correct assembly of RNAP I, II, and III in an Rpb5p-dependent manner	+	+	+
*CCR4* (1,3,4)	Component of the CCR4-NOT transcriptional complex. CCR4-NOT is involved in regulation of gene expression. Ccr4p is a component of the major cytoplasmic deadenylase, which is involved in mRNA poly(A) tail shortening	+	+	+
*CDC73* (1,4,5)	Component of the Paf1 complex. Cdc73p binds to and modulates the activity of RNA polymerases I and II. Cdc73p is also required for expression of certain genes, modification of some histones, and telomere maintenance. It is also involved in transcription elongation	0	+	+
*CSE2* (2,3,4)	Subunit of the RNA polymerase II mediator complex, required for regulation of RNA polymerase II activity. Cse2p associates with core polymerase subunits to form the RNA polymerase II holoenzyme	+	+	+
*CTK1* (1,4,5)	Catalytic (alpha) subunit of C-terminal domain kinase I (CTDK-I). Ctk1p phosphorylates both RNA pol II subunit Rpo21p to affect transcription and pre-mRNA 3' end processing, and ribosomal protein Rps2p to increase translational fidelity	+	+	+
*DST1* (1,2,4)	General transcription elongation factor TFIIS. Dst1p enables RNA polymerase II to read through blocks to elongation by stimulating cleavage of nascent transcripts stalled at transcription arrest sites. Dst1p also maintains RNAPII elongation activity on ribosomal protein genes during conditions of transcriptional stress	0	+	0
*GAL11* (2,3,4)	Subunit of the RNA polymerase II mediator complex that affects transcription by acting as target of activators/ repressors. Gal11p associates with core polymerase subunits to form the RNA polymerase II holoenzyme	+ +	+ +	+ +
*HMO1* (2,5,6)	Chromatin associated high mobility group (HMG) family member, playing a role in genome maintenance. Hmo1p is involved in compacting, bending, bridging and looping DNA. Also, Hmo1p is a rDNA-binding component that regulates transcription from RNA polymerase I promoters. Hmo1p also regulates start site selection of ribosomal protein genes via RNA polymerase II promoters	+ +	+	+
*HPR*1 (1,5)	Subunit of THO/TREX complexes and a subunit of an RNA Pol II complex. Hpr1p regulates lifespan and it is also involved in telomere maintenance	+	+	+
*MED1* (3,4)	Subunit of the RNA polymerase II mediator complex. Med1p associates with core polymerase subunits to form the RNA polymerase II holoenzyme, being essential for transcriptional regulation	+	+	+
*MED2* (2,3,4)	Subunit of the RNA polymerase II mediator complex. Med2p associates with core polymerase subunits to form the RNA polymerase II holoenzyme, being essential for transcriptional regulation	+	+ +	+ +
*NUP133 *(3,4,6)	Subunit of Nup84 subcomplex of nuclear pore complex (NPC). Nup133p contributes to nucleocytoplasmic transport, NPC biogenesis. It is also involved in double-strand break repair, transcription and chromatin silencing	+	+	+
*NUP84* (4,6)	Subunit of the Nup84 subcomplex of the nuclear pore complex (NPC). Nup84p contributes to nucleocytoplasmic transport and NPC biogenesis. It is also involved in double-strand break repair, transcription and chromatin silencing	+	+	+
*POP2* (1,3,4)	Subunit of Ccr4-Not complex that mediates 3' to 5' mRNA deadenylation	+	+	+
*REG1* (4,6)	Regulatory subunit of type 1 protein phosphatase Glc7p. Reg1p is involved in negative regulation of glucose-repressible genes and in the regulation of nucleocytoplasmic shuttling of Hxk2p	+	+	+
*RIC1* (4,6)	Protein involved in retrograde transport to the cis-Golgi network and in the transcription of rRNA and ribosomal protein genes	+	+	+
*RPB4* (2,3)	RNA polymerase II subunit B32 that forms a dissociable heterodimer with Rpb7p. Rpb4/7 regulates cellular lifespan via mRNA decay process. Rpb4 is involved in recruitment of 3'-end processing factors to transcribing RNAPII complex, export of mRNA to cytoplasm under stress conditions; also involved in translation initiation	+	+ +	+
*SIN3 *(4,5)	Component of both the Rpd3S and Rpd3L histone deacetylase complexes. Sin3p is involved in transcriptional repression and activation of diverse processes (e.g., mating-type switching and meiosis). It is also involved in the maintenance of chromosomal integrity	0	+	+
*SIN4* (2,3,4)	Subunit of the RNA polymerase II mediator complex. Sin4p associates with core polymerase subunits to form the RNA polymerase II holoenzyme. Sin4 contributes to both positive and negative transcriptional regulation	+	+ +	+
*SNF2* (4,6)	Catalytic subunit of the SWI/SNF chromatin remodeling complex. Snf2p contains DNA-stimulated ATPase activity involved in transcriptional regulation	+ +	+	+
*SNF6* (4,5,6)	Subunit of the SWI/SNF chromatin remodeling complex, involved in transcriptional regulation	+	+	+ +
*SPT4* (1,3,4,5,6)	Spt4p/5p (DSIF) transcription elongation factor complex subunit. The Spt4/5 complex binds to ssRNA in a sequence-specific manner, and along with RNAP I and II has multiple roles regulating transcriptional elongation, RNA processing, quality control, and transcription-coupled repair. Spt4p influences chromosomal dynamics and silencing	+	+ +	+
*SRB5* (2,4)	Subunit of the RNA polymerase II mediator complex. Srb5p associates with core polymerase subunits to form the RNA polymerase II holoenzyme. It is essential for transcriptional regulation and required for proper termination of transcription for some genes and for telomere maintenance	+ +	+	+
*SRB8* (3,4)	Subunit of the RNA polymerase II mediator complex. Srb8p associates with core polymerase subunits to form the RNA polymerase II holoenzyme Srb8p is essential for transcriptional regulation and it is also involved in glucose repression	+	+	0
*SUB1*(1,4)	Transcriptional regulator. Subp1 facilitates elongation through factors that modify RNAP II. It also plays a role in nonhomologous end-joining (NHEJ) of double-strand breaks in plasmid DNA and in the hyperosmotic stress response through polymerase recruitment at RNAP II and RNAP III genes. Sub1p negatively regulates sporulation	+	+	0
*SWI3* (4,6)	Subunit of the SWI/SNF chromatin remodeling complex. SWI/SNF regulates transcription by remodeling chromosomes. Swi3p contains SANT domain that is required for SWI/SNF assembly and it is essential for displacement of histone H2A-H2B dimers during ATP-dependent remodeling	+	+	0
*TAF14* (2,6)	Subunit of TFIID, TFIIF, INO80, SWI/SNF, and NuA3 complexes. Taf14p is involved in RNA polymerase II transcription initiation and in chromatin modification	+	+	+
*THO2* (1,5)	Subunit of the THO complex; THO is required for efficient transcription elongation and involved in transcriptional elongation-associated recombination	+	+	+ +
*TUF1* (3,4,6)	Mitochondrial translation elongation factor Tu (EF-Tu). Tuf1p is involved in fundamental pathway of mtDNA homeostasis. Tuf1p comprises both GTPase and guanine nucleotide exchange factor activities	+	+	+
*TUP1* (3,4,6)	General repressor of transcription in complex with Cyc8p. Tup1p is involved in the establishment of repressive chromatin structure through interactions with histones H3 and H4 and stabilization of nucleosomes over promoters	+ +	+ +	+ +
*VPS34* (1,4)	Phosphatidylinositol (PI) 3-kinase that synthesizes PI-3-phosphate. Vps34p forms membrane-associated signal transduction complex with Vps15p to regulate protein sorting	+ +	+ +	+
*XRN1* (1,3,4)	Evolutionarily-conserved 5'-3' exonuclease and deNADding enzyme that modulates mitochondrial NAD-capped RNA. Xrn1p is involved in mRNA decay. It positively regulates transcription initiation and elongation involved in ribosomal RNA maturation, telomere maintenance, and turnover of tRNA introns. Xrn1p is also a negative regulator of autophagy	+	+	+

The table includes the list of genes, involved in yeast transcription, identified in this study as determinants of yeast tolerance to acetic acid (C2), butyric acid (C4), or octanoic acid (C8). Table elaborated as described in Table 1

(1) “Positive regulation of DNA-templated transcription, elongation” (C2/C4/C8)

(2) “Transcription initiation at RNA polymerase II promoter” (C2/C4)

(3) “Negative regulation of macromolecule biosynthetic process” (C2)

(4) “Positive regulation of transcription by RNA polymerase II” (C2/C4)

(5) “Regulation of transcription by RNA polymerase I” (C4)

(6) “Chromatin remodeling” (C2)

Ten transcription factors were identified, and their specific regulatory functions are described in Table 4. Seven of these transcription factors (*CBF1, DAL81, GCR2, MGA2, RPN4, STP1* and *UME6*) are required for tolerance to the three weak acids while *RPH1* is exclusive of the octanoic acid dataset, and *ROX1* and *SFP1* are exclusive of the butyric acid dataset. The Yeastract + database [41] was used to retrieve the number of tolerance genes present in each dataset that are described as regulated by the identified transcription factors and the percentage of transcription factor-regulated genes present in each dataset is highly variable **(**Table 4**)**.

**Table 4:** Percentage of tolerance genes in each dataset regulated by transcription factors identified as tolerance determinants

Gene/ORF	Description of the encoded protein function	Susceptibility level and percentage of tolerance genes present in each dataset that are regulated by TFs also identified as tolerance determinants		
		C2	C4	C8
*CBF1*	Transcription factor associated with kinetochore proteins; required for chromosome segregation	+ /76.6	+ /78.0	+ /76.9
*DAL81*	Transcription factor acting as a positive regulator of genes involved multiple nitrogen degradation pathways	+ /4.5	+ /4.7	+ /4.7
*GCR2*	Transcriptional activator of genes involved in glycolysis	+ /15.8	+ /15.4	+ /16.3
*MGA2*	Transcription factor involved in regulation of *OLE1* transcription	+ /16.8	+ / 14.2	+ /12.4
*ROX1*	Transcription factor repressing hypoxic genes in aerobic conditions	0	+ /15.6	0
*RPH1*	Transcription factor involved in the repression of autophagy-related genes in nutrient-replete conditions	0	0	29.4
*RPN4*	Transcription factor that activates the transcription of proteasome encoding genes being regulated by the 26S proteasome in a negative feedback control mechanism	+ /83.2	+ /86.0	+ /85.7
*SFP1*	Transcription factor that regulates ribosomal protein and biogenesis genes; also involved in the regulation of the response to nutrients and stress, G2/M transition during mitotic cell cycle and DNA-damage response and modulates cell size	0	49.3	0
*STP1*	Transcription factor that activates transcription of amino acid permease genes and may have a role in tRNA processing	+ /13.4	+ /11.8	+ /11.2
*UME6*	Transcriptional regulator of early meiotic genes; involved in chromatin remodeling and transcriptional repression via DNA looping	+ /15.3	+ /16.6	+ /15.5

Clustering of tolerance genes toward acetic acid (C2), butyric acid (C4), or octanoic acid (C8) found to be targets of transcription factors that are also determinants of tolerance to each weak acid. Information was retrieved from the Yeastract + database (November 2022)

Table elaborated as described in Table 1

### Genes involved in protein synthesis

The translation process, both cytoplasmic and mitochondrial, was found to be enriched in the datasets obtained for the three weak acids. The “Cytoplasmic translation” function includes genes (complete list in Additional file 14: Table S14) that encode proteins of the small and large ribosomal subunits, in general identified as *RPS* genes (Ribosomal Proteins of the Small subunit)—(e.g., *RPS11A, RPS19B, RPS21B* and *RPS30B*)—and *RPL* genes (Ribosomal Proteins of the Large subunit), respectively—(e.g., *RPL12B, RPL13B, RPL21A, RPL31A* and *RPL39*). Other genes involved in cytoplasmic translation as, for example, *BUD27* and *TIF3,* are involved in translation initiation. Genes involved in “Mitochondrial translation” (complete list in Additional file 15: Table S15) encode proteins of the mitochondrial ribosomal small subunit (*MRPS* or *RSM* genes)—(e.g., *MRPS12*, *RSM23* and *RSM7*)—and of the mitochondrial ribosomal large subunit (*MRPL* genes)—(e.g., *MRPL15*, *MRPL23, MRPL33* and *MRPL49*). Other genes related to mitochondrial translation and required for tolerance to acetic, butyric and octanoic acids are *SLM5* and *TUF1*, involved in tRNA synthesis and translational elongation, respectively.

### Genes involved in mitochondrial genome maintenance

The maintenance of mitochondrial DNA level and integrity is essential for a functional respiratory function [42] and the function “Mitochondrial genome maintenance” was exclusively enriched in the octanoic acid dataset. Considering the more marked effect of lipophilic fatty acids, such as octanoic acid, in mitochondrial membrane permeability [43], it is likely that the action of octanoic acid in yeast cells may target mitochondrial function, triggering different cellular responses, such as mitochondrial genome maintenance or mitochondrial fusion to counteract these perturbations [44]. *MGM1*, a gene coding for a dynamin-related large GTPase required for inner membrane fusion [45] is among the twelve identified genes with a role in mitochondrial genome maintenance: *EXO5, ILM1, IRC3, MDJ1, MDM12, MGM1, MHR1, MIP1, MRPL51, RRG1, RRG8* and *RRG9* **(**Table 5**)**. Two of them, (*MHR1* and *MRPL51*, are also involved in “Mitochondrial Translation”, *RRG1* encodes a protein that also appears to play a role in vacuolar acidification **(**Table 2**)** and the other five genes (*EXO5, MDM12, MIP1, RRG8* and *RRG9*) are exclusive to the octanoic acid dataset.

**Table 5:** Genes involved in mitochondrial genome maintenance

Gene/ ORF	Description of the encoded protein function	Susceptibility		
		C2	C4	C8
*EXO5*	Mitochondrial 5′-3′ exonuclease and sliding exonuclease required for mitochondrial genome maintenance	0	0	+
*ILM1*	No demonstrated function but Ilm1p may be involved in mitochondrial DNA maintenance	+	+	+
*IRC3*	Double-stranded DNA-dependent helicase of the DExH/D-box family, also containing double-stranded DNA translocase activity. Irc3p is responsible for maintenance of the mitochondrial (mt) genome	0	+	+
*MDJ1*	Co-chaperone that stimulates HSP70 protein Ssc1p ATPase activity. Mdj1p participates in protein folding/refolding in the mitochodrial matrix	+ +	+ +	+ +
*MDM12*	Mitochondrial outer membrane protein, ERMES complex subunit. Mdm12p is required for transmission of mitochondria to daughter cells and mitophagy	0	0	+
*MGM1*	Mitochondrial GTPase, present in a complex with Ugo1p and Fzo1p. Mgm1p is required for mitochondrial morphology, fusion, and genome maintenance	+	+	+
*MHR1*	Mitochondrial ribosomal protein of the large subunit. Mhr1p is involved in homologous recombination in mitochondria; it is required for recombination-dependent mtDNA partitioning; and stimulation of mitochondrial DNA replication in response to oxidative stress	0	+	+
*MIP1*	Mitochondrial DNA polymerase gamma	0	0	+
*MRPL51*	Mitochondrial ribosomal protein of the large subunit. Mrpl51p is required for mitochondrial genome integrity, respiratory growth, and mitochondrial redox homeostasis	+	+	+
*RRG8*	No demonstrated function but Rrg8p may be required for efficient 5′ processing of mitochondrial tRNAs, for respiratory growth and mitochondrial genome maintenance	0	0	+
*RRG9*	No demonstrated function but *rrg9*Δ lacks mitochondrial DNA and cannot grow on glycerol	0	0	+

The table includes the list of genes involved in mitochondrial genome maintenance, identified in this study as determinants of yeast tolerance to acetic acid (C2), butyric acid (C4) or octanoic acid (C8). Table elaborated as described in Table 1

### Genes involved in ATPase complex assembly

The function “Proton-transporting two-sector ATPase complex assembly” was found to be exclusively enriched in the octanoic acid dataset while the butyric acid dataset is the only dataset significantly enriched in genes related to “Mitochondrial proton-transporting ATP synthase complex assembly”. These two functions, despite being described as separate functions by the PANTHER Classification System [38], collectively include a group of genes related to ATP synthesis by the ATPase complex, which typically occurs at the mitochondrial level. In total, these two functions include 11 genes of our datasets, listed in Table 6 (*ATP10, ATP11, ATP12, ATP22, ATP23, ATP25, OXA1, PFK2, PKR1, VMA21, VPH2*), five of which (*ATP10, ATP11, ATP23, ATP25, OXA1*) are shared between the two functions. Two of them, related to “Proton-transporting two-sector ATPase complex assembly (*PFK2* and *VPH2*) and present in all three datasets, are also considered in the “Vacuolar acidification” function. This suggests that the ATPase complex assembly is also part of a common stress response with emphasis on octanoic acid, since the octanoic acid dataset includes 10 of the 11 genes listed in Table 6.

**Table 6:** Genes involved in the ATPase complex assembly

Gene/ORF	Description of the encoded protein function	Susceptibility		
		C2	C4	C8
*ATP10* (1,2)	Assembly factor for the F0 sector of mitochondrial F1F0 ATP synthase	0	+	+
*ATP11* (1,2)	Molecular chaperone. Atp11p is required for the assembly of alpha and beta subunits into the F1 sector of mitochondrial F1F0 ATP synthase	0	+	+
*ATP12* (2)	Assembly factor for F1 sector of mitochondrial F1F0 ATP synthase. Atp12p is required for assembly of alpha and beta subunits into F1 sector of mitochondrial F1F0 ATP synthase	+	+	0
*ATP22* (1)	Specific translational activator for the mitochondrial ATP6 mRNA	0	0	+ +
*ATP23*(1,2)	Putative metalloprotease of the mitochondrial inner membrane, required for processing of Atp6p. Also, Atp23p has an additional role in assembly of the F0 sector of the F1F0 ATP synthase complex	0	+	+
*ATP25* (1, 2)	Protein that associates with mitochondrial ribosome. Atp25p is required for the stability of Oli1p (Atp9p) mRNA	+	+	+
*OXA1* (1,2)	Mitochondrial inner membrane insertase. Oxa1p mediates the insertion of both mitochondrial- and nuclear-encoded proteins from the matrix into the inner membrane and plays a role in the insertion of carrier proteins into the inner membrane. Also, Oxa1p acts as a voltage-gated ion channel, activated by substrate peptides	+	+	+
*PFK2* (1)	Beta subunit of heterooctameric phosphofructokinase, involved in glycolysis	+ +	+ +	+ +
*PKR1*(1)	V-ATPase assembly factor. Pkr1p functions with other V-ATPase assembly factors in the ER to efficiently assemble the V-ATPase membrane sector (V0)	0	0	+
*VMA21* (1)	Integral membrane protein required for V-ATPase function. Vma21p is not an actual component of the vacuolar H+-ATPase (V-ATPase) complex.)	0	+	+
*VPH2*(1)	Integral membrane protein required for V-H^+^-ATPase function. Vph2p is involved in the assembly of V-H^+^-ATPase	+	+	+

The table includes the list of genes involved in the ATPase complex assembly**,** identified in this study as determinants of yeast tolerance to acetic acid (C2), butyric acid (C4) or octanoic acid (C8). Table elaborated as described in Table 1

(1) “Proton-transporting two-sector ATPase complex assembly” (C8)

(2) “Mitochondrial proton-transporting ATP synthase complex assembly” (C4)

### Genes involved in yeast cell wall synthesis and assembly

The yeast cell wall is an essential organelle required for cell’s structural integrity and the first barrier to withstand a wide range of stressors [12, 46, 47]. Of the extensive list of genes involved in cell wall synthesis and remodeling [48], 11 genes (*CHS1, CTS1, FKS1, GAS1, HOC1, KRE6, MNN10, MNN2, MNN9, OCH1* and *YGP1*) were identified as being determinants of tolerance to acetic acid, butyric acid or octanoic acid (Table 7). Five of these 11 genes were found in the three datasets and the deletion of three of these genes (*MNN10, MNN9 and OCH1*) resulted in full growth inhibition (+ +) under stress induced by the three acids. We also found that nine of the 11 genes are determinants of octanoic acid tolerance, while the number for acetic acid (seven genes) and butyric acid (six genes) is lower (Table 7**).** As demonstrated before for acetic acid [47, 49], these results are consistent with the important role of the cell wall in the response and tolerance to these weak acids, especially to the more liposoluble acid.

**Table 7:** Genes involved in cell wall synthesis and assembly

Gene/ORF	Description of the encoded protein function	Susceptibility		
		C2	C4	C8
*CHS1*	Chitin synthase I required for repairing the chitin septum during cytokinesis	+	0	0
*CTS1*	Endochitinase required for cell separation after mitosis; transcriptional activation mediated by transcription factor Ace2p	0	+	0
*FKS1*	Catalytic subunit of 1,3-beta-D-glucan synthase; functionally redundant with alternate catalytic subunit Gsc2p. Fks1p binds to regulatory subunit Rho1p and is involved in cell wall synthesis and maintenance, localizing to sites of cell wall remodeling	+	+	+
*GAS1*	Beta-1,3-glucanosyltransferase required for cell wall assembly. Gas1p interaction with histone H3 lysine acetyltransferases *GCN5* and *SAS3* indicate a role in DNA damage response and cell cycle regulation	+	+	+
*HOC1*	Alpha-1,6-mannosyltransferase involved in cell wall mannan biosynthesis	+	0	+
*KRE6*	Glucosyl hydrolase required for beta-1,6-glucan biosynthesis	0	0	+
*MNN10*	Subunit of a Golgi mannosyltransferase complex that mediates elongation of the polysaccharide mannan backbone	+ +	+ +	+ +
*MNN2*	Alpha-1,2-mannosyltransferase responsible for addition of the first alpha-1,2-linked mannose to form the branches on the mannan backbone of oligosaccharides	0	0	+
*MNN9*	Subunit of Golgi mannosyltransferase complex that mediates elongation of the polysaccharide mannan backbone. Separately forms a complex with Van1p that is also involved in backbone elongation	+ +	+ +	+ +
*OCH1*	Mannosyltransferase of the cis-Golgi apparatus involved in initiating the polymannose outer chain elongation of N-linked oligosaccharides of glycoproteins	+ +	+ +	+ +
*YGP1*	Cell wall-related secretory glycoprotein that is induced by nutrient deprivation-associated growth arrest and upon entry into stationary phase	0	0	+

The table includes the list of genes involved in yeast cell wall synthesis and assembly, identified in this study as determinants of yeast tolerance to acetic acid (C2), butyric acid (C4) or octanoic acid (C8). Table elaborated as described in Table 1

### Genes involved in lipid biosynthesis and distribution within the membranes

Among the three datasets, 24 genes were identified as involved in biosynthesis and spatial organization of phospholipids within the membranes (eight genes—*CDC50, CHO1, DNF2, DRS2, GEP4, LEM3, OPI3* and *PSD1*), sphingolipids (seven genes—*CSG2, ELO2, ELO3, SAC1, SKN1, SUR1* and *SUR2*) and sterols (six genes—*ERG2, ERG24, ERG28, ERG4, ERG5* and *LAF1*) (Table 8).A full growth inhibition was associated with the deletion of five genes (*CDC50, ERG2, ERG28, ERG4* and *SAC1*), four genes (*ERG2, ERG28, ERG4* and *SAC1*), and seven genes (*CSG2, DNF2, ELO2, ERG2, ERG28, SPF1, SUR1*), after exposure to acetic acid, butyric acid or octanoic acid, respectively. These results are consistent with the reported occurrence of alterations in the biosynthesis of phospholipids, sphingolipids and sterols upon exposure to weak acids [23, 24, 49, 50]. They also suggest a higher impact of octanoic acid on cellular membranes.

**Table 8:** Genes involved in lipid biosynthesis and distribution within the membranes

Gene/ORF	Description of the encoded protein function	Susceptibility		
		C2	C4	C8
*CDC50*	Endosomal protein that interacts with phospholipid flippase Drs2p	+ +	0	0
*CHO1*	Phosphatidylserine synthase required for phospholipid biosynthesis	0	+	+
*CSG2*	Endoplasmic reticulum membrane protein required for mannosylation of inositolphosphorylceramide Csg2p concentration increases in response to DNA replication stress	0	+	+ +
*DNF2*	Aminophospholipid translocase (flippase) involved in phospholipid translocation, contributing to endocytosis, protein transport, and cellular polarization	0	0	+ +
*DRS2*	Trans-golgi network aminophospholipid translocase (flippase) that maintains membrane lipid asymmetry in post-Golgi secretory vesicles and contributes to clathrin-coated vesicle formation, endocytosis, protein trafficking between the Golgi and endosomal system and the cellular response to mating pheromone	+	+	+
*ELO2*	Fatty acid elongase that is involved in sphingolipid biosynthesis and acts on fatty acids of up to 24 carbons in length	+	+	+ +
*ELO3*	Elongase involved in fatty acid and sphingolipid biosynthesis, synthesizing very long chain 20–26-carbon fatty acids from C18-CoA primers. Elo3p is also involved in regulation of sphingolipid biosynthesis	+	0	+
*ERG2*	C-8 sterol isomerase that catalyzes isomerization of delta-8 double bond to delta-7 position at an intermediate step in ergosterol biosynthesis. *ERG2* expression is down-regulated when ergosterol is in excess	+ +	+ +	+ +
*ERG24*	C-14 sterol reductase that acts in ergosterol biosynthesis	+	+	+
*ERG28*	Endoplasmic reticulum membrane protein that possibly facilitates protein–protein interactions between the Erg26p dehydrogenase and the Erg27p 3-ketoreductase and/or tether these enzymes to the ER, also interacts with Erg6p	+ +	+ +	+ +
*ERG4*	C-24(28) sterol reductase that catalyzes the final step in ergosterol biosynthesis	+ +	+ +	0
ERG5	C-22 sterol desaturase. Erg5p is a cytochrome P450 enzyme that catalyzes the formation of the C-22(23) double bond in the sterol side chain in ergosterol biosynthesis	+	0	+
*FAA3*	Long chain fatty acyl-CoA synthetase that activates imported fatty acids with a preference for C16:0-C18:0 chain lengths	0	0	+
*GEP4*	Mitochondrial phosphatidylglycerophosphatase that dephosphorylates phosphatidylglycerolphosphate to generate phosphatidylglycerol, an essential step during cardiolipin biosynthesis	+	+	+
*LAF1*	Sterol-binding beta-propeller protein essential for retrograde transport of ergosterol from the plasma membrane to the endoplasmic reticulum	0	0	+
*LEM3*	Membrane protein of the plasma membrane and ER that interacts specifically in vivo with the phospholipid translocase (flippase) Dnf1p. Lem3p is involved in translocation of phospholipids and alkylphosphocholine drugs across the plasma membrane	+	0	0
*LOA1*	Lysophosphatidic acid acyltransferase involved in triacelglyceride homeostasis and lipid droplet formation	+	+	+
*OPI3*	Methylene-fatty-acyl-phospholipid synthase that catalyzes the last two steps in phosphatidylcholine biosynthesis. Opi3p is also known as phospholipid methyltransferase	+	0	+
*PSD1*	Phosphatidylserine decarboxylase of the mitochondrial inner membrane, converts phosphatidylserine to phosphatidylethanolamine	0	0	+
*SAC1*	Phosphatidylinositol phosphate phosphatase with a role in protein trafficking, processing, secretion, and cell wall maintenance. Sac1p also regulates sphingolipid biosynthesis	+ +	+ +	0
*SKN1*	Protein involved in sphingolipid biosynthesis	0	0	+
*SPF1*	P-type ATPase required to maintain normal lipid and sterol composition of intracellular compartments. Spf1p is also involved in Ca^2+^ homeostasis	0	0	+ +
*SUR1*	Mannosylinositol phosphorylceramide synthase catalytic subunit that forms a complex with regulatory subunit Csg2p	0	0	+ +
*SUR2*	Sphinganine C4-hydroxylase, catalyses the conversion of sphinganine to phytosphingosine in sphingolipid biosynthesis	+	0	0

The table includes the list of genes, involved in lipid biosynthesis and distribution within the membranes**,** identified in this study as determinants of yeast tolerance to acetic acid (C2), butyric acid (C4) or octanoic acid (C8). Table elaborated as described in Table 1

### Genes encoding membrane transporters

Ten genes encoding membrane transporters (*BAP2, GUP1, OXA1, PDR12, PET8, PMR1, POR1, PRM6, TAT1* and *TRK1*) (Table 9) are present in the three datasets. All proton pumps, mainly vacuolar proton translocating ATPases, encoded by *VMA* genes, were not considered in Table 9 since they were included in other functional groups already described.

**Table 9:** Genes encoding membrane transporters

Gene/ORF	Description of the encoded protein function	Susceptibility		
		C2	C4	C8
*BAP2*	High-affinity leucine permease. Bap2p is also involved in the uptake of leucine, isoleucine and valine	+	+	0
*GUP1*	Plasma membrane protein involved in remodeling GPI anchors. Gup1p is proposed to be involved in glycerol transport	0	+	+
*OXA1*	Mitochondrial inner membrane insertase that also acts as a voltage-gated ion channel	+	+	+
*PDR12*	Plasma membrane ATP-binding cassette (ABC) transporter that is a weak-acid-inducible multidrug transporter required for weak organic acid resistance	0	+ +	+
*PET8*	S-adenosylmethionine transporter of the mitochondrial inner membrane	0	+	+
*PMR1*	High affinity Ca^2+^/Mn^2+^ P-type ATPase required for Ca^2+^ and Mn^2+^ transport into Golgi	+	+	+
*POR1*	Mitochondrial porin (voltage-dependent anion channel)	0	+	+
*PRM6*	Potassium transporter that mediates K^+^ influx and activates the high-affinity Ca^2+^ influx system	0	+	0
*TAT1*	Amino acid transporter for valine, leucine, isoleucine, and tyrosine with low-affinity for tryptophan and histidine	0	0	+
*TRK1*	Component of the Trk1p-Trk2p potassium transport system. Trk1p is a high affinity potassium transporter	+	+	+

The table includes the list of genes, encoding membrane transporters**,** identified in this study as determinants of yeast tolerance to acetic acid (C2), butyric acid (C4) or octanoic acid (C8). Table elaborated as described in Table 1

Among the yeast transporters required for multidrug resistance (MDR), only *PDR12* was identified in the datasets, and only for the more lipophilic acids datasets (butyric and octanoic acids). This transporter was already on the focus of several studies [19, 23, 51, 52] and is considered essential for tolerance to weak acids with medium carbon chain. Bap2p, Pet8p and Tat1p are amino acid transporters, in line with indications for an increased protein synthesis under stress and the registered enrichment of genes involved in “Cytoplasmic translation” and “Mitochondrial translation” in the datasets. Consistent with the major role of potassium availability in acetic acid tolerance mechanisms [11, 53], the *TRK1* gene, encoding the high-affinity potassium plasma membrane potassium transporter that mediates K^+^ influx, was identified, and for the three datasets. Another potassium transporter that mediates K^+^ influx and activates the high-affinity Ca^2+^ influx system, Prm6p, was found to confer tolerance to butyric acid.

## Discussion

To provide the indispensable holistic understanding of the common and the more specific molecular determinants and biological functions underlying tolerance to three mono-carboxylic linear weak acids of different lipophilicity and relevance in the context of sustainable biotechnology, a chemogenomic analysis complemented by physiological studies, was performed. Given the objective, the study design had in due consideration the importance of using equivalent inhibitory sublethal concentrations of the weak acids to be compared. Under such conditions, it was found that the number of the tolerance genes identified increased with the weak acid chain length. A substantial overlap was found, and 268 genes were identified as tolerance determinants for the three acids. Among these genes, many are related with intracellular trafficking, including late endosome to vacuole transport, endosomal transport, protein targeting/Localization to vacuole and ubiquitin-dependent protein catabolic process via the Multivesicular Body (MVB) sorting pathway. Several *VPS* and *PEP* groups of genes were identified as acetic, butyric and octanoic acids tolerance determinants; they are involved, among other functions, in the recognition of localization signals and sorting and transport of proteins to the vacuole [54]. These categories were also found to be enriched in previous genome-wide studies, namely in ethanol, formic acid and other food-relevant stresses [16, 27, 55], highlighting their role in yeast stress tolerance.

Vacuolar acidification was classified as an enriched function under the tested conditions, with 17 genes identified, most of them encoding subunits of the multimeric vacuolar H^+^-ATPase or required for its assembly. The activity of the yeast vacuolar- ATPase (V-ATPase) is involved in the maintenance of intracellular pH (pHi) [56], a highly controlled physiological parameter within cells whose regulation is critical for normal functioning, from energy generation to protein folding and activity [57]. Several mechanisms of pHi regulation are known to be induced under weak acid stress allowing pHi recovery up to more physiological values, following the dissociation of weak acids in the cytosol and the increase of plasma membrane permeability induced by the liposoluble acid form [58]. These mechanisms include the activation of plasma membrane H^+^-ATPase activity. Under weak acid stress, the activation of plasma membrane H^+^‐ATPase activity is considered among the major tolerance mechanisms of response to weak acids, in particular to acetic acid and octanoic acid [11, 36, 59–63]. However, such activation implicates higher energy consumption and, even in the absence of stress, this H^+^-pump is the major ATP consumer in the cell, consuming up to 20% of cellular ATP in actively growing cells in glucose [64]. Since the deletion mutant collection used in this work does not include essential genes, the major form of the plasma membrane H^+^-ATPase encoded by the essential *PMA1* gene could not be identified. According to our results, when yeast cells were suddenly exposed to equivalent and moderate inhibitory concentrations of acetic, butyric and octanoic acids, a deep and rapid decrease of pHi was observed but, presumably, as the result of the induction of adaptation mechanisms to counteract this intracellular acidification, pHi recover to more physiological levels but could not reach unstressed cells pHi. Results also suggest that for the level of weak acid stress examined, intracellular acidification cannot be considered a major toxicity mechanism and do not indicate a marked difference among the moderate effect of the three weak acids tested, consistent with the occurrence of in common efficient mechanisms of response at this level.

Genes involved in autophagic processes, in particular macroautophagy, were also shown to be required for yeast tolerance to the three weak acids examined. Macroautophagy involves the formation of double-membrane that fuse with the vacuole, resulting in delivery and subsequent degradation of the cargo in the interior of this organelle [65]. Autophagy-related family of genes (*ATG* genes), as the name suggests, is related to autophagy and autophagy-related processes [66]. *ATG20,* a sorting nexin required for the cytoplasm-to-vacuole targeting (Cvt) pathway, endosomal sorting, and selective autophagy [67], was found to confer tolerance to the three acids. However, *ATG11,* an adapter protein with a fundamental role in selective macroautophagy, coordinating several steps of selective autophagy from cargo selection and phagophore assembly site organization to autophagosome maturation and the termination of selective autophagy [68], was identified exclusively in octanoic acid dataset*.* The degradation of ribosomes, also called ribophagy [69] was also found to be important in the context of tolerance to acetic, butyric, and octanoic acids. Pro1p, a gamma-glutamyl kinase required for nitrogen starvation-induced ribophagy [70] was identified as a tolerance determinant in the three datasets and the growth of the corresponding deletion mutant, *pro1Δ*, was abrogated under the tested conditions, corroborating the high sensitivity already reported for this mutant under various stresses [71]. The *PRO1* gene genetically interacts with *BRE5,* an ubiquitin protease cofactor that is part of the Bre5-Ubp3 complex required for deubiquitination activity in ribophagy [72]. *BRE5* and *UBP3* were exclusively found in butyric acid and octanoic acid datasets. Under nitrogen-starvation, ribophagy was hypothesized to avoid the production of unnecessary proteins and/or to provide amino acid for new protein synthesis under autophagy-triggering (nitrogen-starvation) conditions [70]. Remarkably, our results also indicate the enrichment of a group of genes involved in “protein synthesis”, mostly in cytoplasmic and mitochondrial translation. The balance between ribophagy and protein synthesis under acetic, butyric, and octanoic acids induced stress appears to be tightly regulated, although not completely understood.

Although glucose, the sole carbon and energy source present in the growth medium, was fully consumed when the stationary phase was attained, the final biomass concentration produced under weak acid stress was below the biomass attained in its absence. This effect was more marked for the more lipophilic weak acids, octanoic acid, followed by butyric acid, indicating a higher dissipation of ATP under stress induced by the more lipophilic acids, as the result of the diversion of ATP for energy-dependent response mechanisms to their deleterious effects. Mitochondria produce the cellular ATP necessary for cellular survival and functioning**,** hosting the tricarboxylic acid (TCA) cycle and oxidative phosphorylation [73, 74]. Besides their role in energy generation, mitochondria are involved in other metabolic processes like amino acid and lipid metabolism and the synthesis of iron–sulfur clusters and heme [73, 74]. In this study, biological functions related to mitochondria, including the assembly of the proton transporting two sector ATPase and mitochondrial genome maintenance were found to be enriched, particularly in octanoic acid-stressed cells**.** Yeast mitochondrial ATP synthase is a rotary molecular machine primarily required for energy generation to be used in cellular processes [75]. This enzyme complex is composed of 17 subunits associated into the soluble F1 sector and a membrane-embedded F0 sector [75]. The individual deletion of genes of the *ATP* family, involved in the assembly of the F1F0 ATP synthase sectors or related with the regulation (activation and processing) of *ATP6* [encoding the subunit a of the F0 sector of mitochondrial F1F0 ATP synthase [75, 76]] led to a susceptibility phenotype to octanoic acid, exclusively. A functional mitochondrial ATP synthase plays a significant role in ATP production for detoxification mechanisms in particular involving the active efflux of the acid counterion out of the cell presumably through the ABC transporter Pdr12p in the specific case of butyrate and octanoate. Pdr12p is already known as responsible for tolerance to acids with medium chain length (e.g. sorbic and propionic acids) but not to short chain length acids (e.g. acetic and formic acids) [51, 52]. This ABC transporter was proposed to be the main responsible for the extrusion of octanoic acid counter-ion accumulated in the cytosol [19].

Concerning mitochondrial respiration, the increase of reactive oxygen species (ROS), including superoxide (O_2_^−^), hydrogen peroxide (H_2_O_2_) and the hydroxyl radical (OH^•^), described as products of aerobic metabolism that are generated when electrons leak from their carrier systems, leading to incomplete reduction of oxygen in a non-enzymatic manner, is a source of membrane damage due to lipid peroxidation [77]. The induction of ROS can damage several cellular molecules and components leading to protein oxidation and oxidative damage of DNA, besides lipid peroxidation. Octanoic acid is suggested to cause oxidative damage to the yeast cell [19] triggering cellular responses involving mitochondria to counteract these perturbations. The first response involves, mitophagy, contributing to mitochondrial homeostasis by preventing the production of excessive ROS [78]. Mitophagy, defined as the degradation of damaged mitochondria [79] was also suggested in our study to play an important role in octanoic acid stress tolerance. Five genes*,* involved in mitophagy and whose deletion was previously reported as causing mitophagy-defective cells [80] were identified in our study for the three weak acids, with *PEP12* being specific to octanoic acid. The second response involves mitochondrial DNA maintenance since mitochondrial DNA is major target for oxidative stress [81]. The integrity of mitochondrial genome has been described as being dependent on mitochondrial fusion and fission [82]. In the octanoic acid dataset, several genes related to mitochondrial fusion and fission were identified further suggesting that tolerance to octanoic acid relies on mitochondrial genome maintenance.

Considering the lipophilic nature of octanoic acid in particular, it can be inferred that mitochondrial damage can also be caused due to the accumulation of this acid in this membrane, as reported for lipophilic compounds [79]. Our results are consistent with the idea that the equivalent inhibitory concentrations of the weak acids tested, in particular of the more lipophilic octanoic acid, affect the organization and function of cellular membranes due to their accumulation in the membranes. The loss of membrane organization and integrity can affect the function of the embedded transport systems and lead to decreased ability to maintain appropriate concentration gradients across membranes [83, 84]. This idea is consistent with the increase of plasma membrane permeability over the first part of exponential growth detected in this study, which was more extensive for octanoic acid, followed by butyric acid-stressed cells. Furthermore, the chemogenomic assay shows that the number of genes present in the octanoic dataset related to lipid biosynthesis and remodeling (19 genes) is substantially higher than the number registered in the butyric (11 genes) and acetic acid (15 genes) datasets. Of the 19 genes present in the octanoic acid dataset, seven are exclusive of this dataset and the deletion of *DNF2, SPF1* or *SUR1* genes result in total inhibition of growth in presence of octanoic acid. *DFN2* and *PSD1* are involved in phospholipid biosynthesis and spatial organization, while *SKN1* and *SUR1* are involved in sphingolipid biosynthesis, *LAF1* is part of ergosterol transport and *FAA3* encodes a long chain fatty acyl-CoA synthetase. The presence of the *FAA3* gene only in the octanoic acid dataset is in line with previous reports demonstrating that the increase of the content of oleic acid in the growth medium leads to a modification of membrane lipid profile accompanied by decreased octanoic acid-induced leakage and increased yeast tolerance [20, 23, 85]. Overall, our data suggests a generalized yeast response involving lipid biosynthesis and remodeling specially when exposed to octanoic acid which is corroborated by other studies that demonstrate an alteration of the plasma membrane lipidic content upon exposure to octanoic acid [23] and that the inhibition of yeast cells by octanoic acid correlates with octanoic acid-induced membrane leakage [23]. The response to acetic acid and butyric acid also involves all types of lipids with a focus on ergosterol and sphingolipids, in line with previous results from our laboratory for acetic acid [47, 49, 50, 84, 86]. We have noticed that deletion of *ERG4* and *SAC1* resulted in total growth inhibition in the presence of acetic and butyric acids, while no susceptibility phenotype was detected for octanoic acid. *SAC1* encodes a lipid phosphatase involved in many cellular processes, such as cell wall maintenance and membrane and protein trafficking, through the regulation of the levels of phosphatidylinositol phosphates, the loss of its activity resulting in decreased sphingolipid synthesis [87, 88]. It has been described that upon exposure to acetic acid there is an increase in Sac1p phosphorylation with a consequent increase in Sac1p activity and promotion of sphingolipid synthesis and leading to enhanced acetic acid tolerance [86, 89]. The impact of acetic acid on the physiology and lipidome of *S. cerevisiae* and *Zygosaccharomyces bailii* was detailed before revealing large lipidomic changes in the highly acetic acid tolerant species *Z. bailii* upon acetic acid exposure, while smaller lipidomic changes were observed in *S. cerevisiae* [89]*.* Therefore, the role of sphingolipids in acetic acid tolerance is well established and was related with the impact that a high content of sphingolipid has in the higher thickness and density of plasma membrane thus increasing the free energy barrier for the permeation of acetic acid through the membrane [90, 91]. Concerning ergosterol, the content of ergosterol is crucial for plasma membrane stability and adequate selective permeability barrier and was found to suffer a drastic reduction in the first hour of cultivation under acetic acid stress [92, 93]. Ergosterol was also related with the formation of lipid-raft domains which may modulate the activity of membrane-embedded pumps required for detoxification [94, 95]. In this context, it is likely that the loss of Erg4p, the final enzyme in the ergosterol biosynthetic pathway, leads to the enhancement of yeast susceptibility to acetic and butyric acids.

The yeast cell wall is a dynamic structure that undergoes major remodeling upon exposure to several stresses, in particular to weak acid stress [96]. Exposure to acetic acid leads to an increase in yeast robustness associated with cell wall increased stiffness and resistance to lyticase activity [47, 49]. The underlying structural modifications are thought to have an impact in avoiding the diffusional re-entry of the non-dissociated form of the weak acid which implicates a futile energy expenditure [47, 49]. In this study eleven genes previously described as part of the cell wall biosynthesis and remodeling pathways [48] were identified as determinants for yeast tolerance to acetic, butyric and/or octanoic acids. The genes *MNN10, MNN9* and *OCH1* encode proteins involved in mannosylation and their deletion caused growth abrogation in presence of the weak acids tested in our study, in line with previous reports [11, 48]. The expression of the *GAS1* gene, encoding a β-1,3-glucanosyltransferase, was described to increase slightly upon exposure to acetic acid, consistent with the defective growth of the deletion mutant in the three datasets [47]. However, *KRE6, MNN2* and *YGP1* genes, previously reported as determinants of tolerance to acetic acid were not found in the acetic acid dataset obtained under mild stressing conditions but were detected for octanoic acid [11, 96]. The *YGP1* gene belongs to the Haa1p regulon, a transcription factor known for its importance in yeast response to acetic acid [97]. The higher number of cell wall-related genes present in the octanoic acid dataset and the fact that approximately 80% of octanoic acid in exposed cells was associated to cell wall fraction [98], suggest the occurrence of a robust response to octanoic acid involving the cell wall, as demonstrated before for acetic acid [47, 49].

Concerning the hypothesized regulatory networks involving acetic acid, butyric acid and octanoic acid tolerance genes, seven TFs were identified in our chemogenomic analysis*. RPH1* is exclusive from the octanoic acid dataset, and *ROX1* and *SFP1* are exclusive of the butyric acid dataset. Concerning Cbf1p and Rpn4p, these TFs reportedly confer tolerance to a wide variety of environmental stresses [99–103]. The regulation data available in the Yeastract database [41] pointed out Cbf1p and Rpn4p as major regulators since more than 75% of the tolerance genes present in the three datasets are known to be under Cbf1p and Rpn4p regulation. Transcription factors (TFs) engineering of promising tolerance targets, either based on the modulation of gene expression [1, 9, 100, 104] or through the alteration of their amino acid sequence [105] is also a potential interesting approach for yeast robustness improvement.

The lists of genes resulting from this genome-wide search, including shared and more specific genes required for maximum tolerance to acetic acid, butyric acid and/or octanoic acids, can be explored for genome manipulation of the yeast cell to obtain more robust strains capable of copying with each weak acid or mixtures of these weak acids. The butyric and octanoic acids datasets are here reported for the first time. This information is useful, not only for the yeast *S. cerevisiae* but also for other biotechnologically relevant species. The exploitation of this new information can be facilitated by the use of the available information and bioinformatics tools in the NCYeastract database (Non- Conventional Yeastract; (http://yeastract-plus.org/ncyeastract/) [41, 106]. In particular, the NCYeastract database is useful for the rapid identification of large lists of orthologous genes involved in tolerance to the weak acids under study in the yeast species currently included in the database. It is also instrumental for the prediction of regulatory associations using the available tools for cross-species transcription regulation comparison [41].

## Conclusions

Results from a chemogenomic analysis and complementary physiological studies revealed toxicity and tolerance mechanisms underlying the action of equivalent moderate inhibitory concentrations of acetic acid, butyric acid and octanoic acid in the yeast model. They also identified genetic determinants and pathways behind yeast tolerance to these weak acids of increasing lipophilicity. Collectively, our results suggest the existence of several basic shared tolerance mechanisms such as vacuolar acidification, intracellular trafficking, autophagy, and protein synthesis, and point to a more marked effect of the more lipophilic acids, specially octanoic acid, on cellular membranes function and lipid remodeling under these stress agents. The importance of a functional mitochondrion, especially for octanoic acid, to provide ATP for energy-dependent detoxification processes is also suggested.

Our findings provided useful lists of genetic determinants and associated tolerance pathways crucial for a better understanding of the toxic action of three monocarboxylic acids of increasing liposolubility. This new information for butyric and octanoic acids is very useful to guide a system biology approach to yeasts of biotechnological relevance to obtain more robust strains for implementation of economically and technologically sustainable bioprocesses towards a circular bio-based economy.

## Methods

### Strains and growth conditions

The haploid parental strain *S. cerevisiae* BY4741 (MATa, *his3*∆*1*, *leu2*∆*0*, *met15*∆*0*, *ura3*∆*0*) and the collection of derived single deletion mutants, obtained from Euroscarf (Frankfurt, Germany), were used for the chemogenomic analysis. For intracellular pH (pHi) assessment and complementary studies, the strain BY4741pHl (his31::loxP-kanMX-loxP-GPD1P-pHluorin) was used [107]. Unless stated otherwise, yeast cells were cultivated at 30 ℃ with orbital agitation (250 rpm) in liquid YPD medium containing, 20 g/L glucose (Merck, Darmstadt, Germany), 10 g/L yeast extract and 20 g/L peptone, both from BD Biosciences (Franklin Lakes, NJ, USA) acidified with HCl until pH 4.5. Solid media were prepared by addition of 20 g/L agar (NZYTech, Lisbon, Portugal).

For pHi assessment, a minimal medium with minimal autofluorescence (MM) was used, containing 20 g/L glucose, 2.67 g/L ammonium sulfate (NH_4_)_2_SO_4_ (Panreac AppliChem, Germany) and 1.9 g/L of YNB (Yeast Nitrogen base without Amino acids, without Ammonium sulphate and without Folic Acid and Riboflavin; Formedium, UK), supplemented with 127.22 mg/L L-glutamic acid (Sigma), 47.28 mg/L L-histidine (Sigma), 110 mg/L leucine (Sigma), 149.92 mg/L L-lysine (Sigma), 40 mg/L methionine (Sigma), 50 mg/L phenylalanine (Sigma), 375 mg/L serine (Sigma), 200 mg/L threonine (Sigma) and 40 mg/L uracil (Sigma), acidified with HCl until pH 4.5. For all experiments, exponentially growing cells were obtained by inoculating fresh medium with yeast cells, cultivated overnight with orbital agitation at 30 ℃. The new culture was incubated in the same conditions until a standardized optical density at 600 nm (OD_600nm_) of 2 was attained.

### Weak acid susceptibility assays in liquid medium

Erlenmeyer flasks with fresh YPD medium at pH 4.5 supplemented or not with equivalent concentrations of acetic acid (62 mM), butyric acid (15.04 mM), or octanoic acid (0.47 mM) were inoculated with exponentially growing BY4741pHl cells. The growth was followed by measuring OD_600 nm_. The equivalent acid concentrations were previously selected following growth in YPD medium, at pH 4.5, supplemented with different concentrations of each acid.

### Assessment of cell viability

The determination of the concentration of viable cells in cell cultures was achieved by calculation of the number of colony forming units (CFU)/mL using the serial dilution method. Yeast cell suspensions were serially diluted in sterile water and 50 μL from selected dilutions were plated in solid YPD medium to obtain between 30 and 300 colonies. Plates were incubated at 30 ℃ for 48 h and colonies were counted to determine CFU/mL. Results are representative of, at least, three independent experiments.

### Assessment of plasma membrane permeability

Plasma membrane permeability was assessed by propidium iodide (PI, Sigma, Germany) staining using flow cytometry in terms of Relative Fluorescence Units (RFUs). Yeast cells growing in YPD medium as described in the “Weak acid susceptibility assays in liquid medium” section. were harvested and suspended to an OD_600 nm_ of 0.6 in phosphate buffered saline [PBS; 1X containing 8 g/L NaCl (Sigma), 2.2 g/L KCl (Sigma), 1.44 g/L Na_2_HPO_4_ (Sigma) and 0.24 g/L KH_2_PO_4_ (Sigma)] and stained for 15 min with 12 μg/mL PI at 30 ℃ with orbital agitation [108]. Flow cytometric analyses were performed using a BD Accuri™ C6 Plus (BD Biosciences). The PI fluorescence was collected via a FL2 585/40 nm filter. A sample of unstained cells was used to define the cell population (R1). As a positive control for cells with maximum permeability, cells were incubated with absolute ethanol at 30 ℃ for 15 min, centrifuged to remove ethanol, and stained with PI as described [108]. The R1 population was divided into two sub-populations. Cells grown under unstressed conditions and stained with PI were used to define a sub-population (M1) and cells treated with ethanol and PI were used to define a complementary sub-population (M2) of PI-positive cells. A fixed total of 50 000 events per sample in M1 were acquired using a slow flow rate (14 μL/min).

### Assessment of glucose consumption

Culture samples, from yeast cells cultures were centrifuged (9700 × g, 3 min) and 100 µL of the supernatant was pipetted into high-performance liquid chromatography (HPLC) vials and diluted with 900 µL of 50 mM H_2_SO_4_. The concentration of glucose present in each sample was determined by HPLC (Hitachi LaChrom Elite, Tokyo, Japan), using a column Aminex HPX- 87H (Bio-Rad, Hercules, CA, USA) coupled with a refractive index detector. Ten microliters of each sample were robotically loaded on the column and eluted with 5 mM H_2_SO_4_ as mobile phase at a flow rate of 0.6 mL/min for 30 min. The column and refractive index detector temperature was set at 65 ℃, respectively. The concentration of glucose was calculated using a calibration curve.

### Assessment of intracellular pH

Intracellular pH (pHi) measurements were made using the BY4741pHl strain that was cultivated as described for susceptibility assays in YPD medium. The method used was adapted from Zimmermannova et al*.* [107]. Briefly, cells were cultivated in MM medium (pH 4.5) supplemented or not with 53 mM acetic acid, 11 mM butyric acid or 0.43 mM octanoic acid. For pHi measurements, fluorescence intensities were recorded at selected timepoints using a FilterMax F5 (Molecular Devices, USA) with an emission filter of 535 nm and excitation filters of 405 and 465 nm. The ratio of emission intensity I405/I465 was used to determine the pHi according to a calibration curve prepared as previously described [109]. For each condition, fluorescence intensities were measured in two wells (200 µL of cells per well; Thermo Scientific™ Nunc MicroWell 96-Well Optical-Bottom Plate, USA). The presented data are average values of at least three independent experiments.

### Genome-wide search for genetic determinants of tolerance to acetic, butyric, and octanoic acids in yeast

To select the equivalent inhibitory acid concentrations to be used for the disruptome assays, the parental strain *S. cerevisiae* BY4741 was tested for susceptibility to a range of acetic acid, butyric acid, or octanoic acid concentrations in YPD solid medium at pH 4.5. Exponentially-growing cell suspensions of BY4741 (OD_600nm_ of 0.5 ± 0.05) were diluted to an OD_600nm_ of 0.25 ± 0.005 (a) and this suspension was used to prepare 1:5 (b), 1:25 (c), 1:125 (d), and 1:625 (e), serially diluted suspensions. Four microliters of each cell suspension were spotted onto YPD solid medium either or not supplemented with acetic acid (70, 80, 90, 100 mM), butyric acid (10, 12, 15, 20 mM) or octanoic acid (0.22, 0.25, 0.30 mM) (results of the screening in the Additional File 16, Figure S1). Susceptibility phenotypes were observed after 48 h of incubation at 30 ℃. To adjust each acid concentration, an additional spot assay was performed based on the previous results, using YPD solid medium supplemented with 75 mM acetic acid, 14 mM butyric acid or 0.30 mM octanoic acid. In this assay, exponentially-growing cell suspensions of BY4741 (OD_600nm_ of 1 ± 0.05) were diluted to an OD_600nm_ of 0.5 ± 0.005 (a) and this suspension was used to prepare 1:2 (b), 1:4 (c), 1:20 (d), 1:100 (e), 1:500 (f), 1:2500 (g) and 1:12500 (h) serially diluted suspensions. Susceptibility phenotypes were observed after 48 h of incubation at 30 ℃. Based on the results of these first screenings, the entire BY4741 Euroscarf deletion mutant collection was screened for susceptibility to the selected equivalent inhibitory concentrations of 75 mM acetic acid, 14 mM butyric acid, or 0.3 mM octanoic acid, at 30 ℃, in YPD medium at pH 4.5. For that, deletion mutant strains were cultivated for 16 h in YPD medium at 30 ℃ with 250 rpm orbital agitation, in 96-well plates. To be used as control, the wild-type strain was prepared individually under the same conditions as the deletion mutants and was inoculated in empty wells, according to the original display of the haploid yeast deletion mutant collection plates. Using a 96-pin replica platter, the cell suspensions were spotted onto the surface of YPD solid medium supplemented, or not, with the selected concentrations and incubated at 30 ℃. Photographs were taken after 24 h of incubation for control plates (YPD medium) or 36–48 h in the presence of the acids.

When observed, the susceptibility phenotype of each single deletion mutant was scored as ( +) if the mutant strain showed, compared with the parental strain, a slight to moderate growth inhibition after the standardized incubation time, and (+ +) if no growth was observed after 48 h of incubation (visual criteria illustrated in the Additional file 17: Figure S2). Only the mutants that exhibited a cell growth in agar plates not supplemented with acid similar to the parental strain were considered for the identification of susceptibility phenotypes.

The eventual over- or under- representation of Gene Ontology (GO) biological process terms related with the physiological function of the genes found to be required for maximum tolerance to acetic acid, butyric acid and octanoic acid was determined using the PANTHER Classification System [38] (http://pantherdb.org). Over-representation of functional categories was considered significant for a p-value < 0.05 and this analysis was complemented using the information available at *Saccharomyces* Genome Database (SGD) [110] (http://www.yeastgenome.org) and at the Yeastract + database [41] (http://www.yeastract.com).

### Supplementary Information

**Additional file 1: Table S1.** List of genes whose expression increases *Saccharomyces cerevisiae* tolerance to 75 mM acetic acid based on the screening of the Euroscarf deletion mutant collection; the elimination of the indicated genes increases yeast susceptibility to acetic acid. The description of the encoded protein functions is based on the information at SGD. The susceptibility phenotype of each single deletion mutant was scored, after 48 h as (+) if the mutant strain showed, compared with the parental strain, a slight to moderate growth inhibition, and (++) if no growth was observed.

**Additional file 2: Table S2.** List of genes whose expression increases *Saccharomyces cerevisiae* tolerance to 14 mM butyric acid based on the screening of the Euroscarf deletion mutant collection; the elimination of the indicated genes increases yeast susceptibility to butyric acid. The description of the encoded protein functions is based on the information at SGD. The susceptibility phenotype of each single deletion mutant was scored, after 48 h as (+) if the mutant strain showed, compared with the parental strain, a slight to moderate growth inhibition, and (++) if no growth was observed.

**Additional file 3: Table S3.** List of genes whose expression increases *Saccharomyces cerevisiae* tolerance to 0.30 mM octanoic acid based on the screening of the Euroscarf deletion mutant collection; the elimination of the indicated genes increases yeast susceptibility to octanoic acid. The description of the encoded protein functions is based on the information at SGD. The susceptibility phenotype of each single deletion mutant was scored, after 48 h as (+) if the mutant strain showed, compared with the parental strain, a slight to moderate growth inhibition, and (++) if no growth was observed.

**Additional file 4: Table S4.** List of tolerance determinants that are exclusive to 75 mM acetic acid dataset.

**Additional file 5: Table S5.** List of tolerance determinants that are exclusive to 14 mM butyric acid dataset.

**Additional file 6: Table S6.** List of tolerance determinants that are exclusive to 0.30 mM octanoic acid dataset.

**Additional file 7: Table S7.** List of tolerance determinants that are shared among 75 mM acetic acid, 14 mM butyric acid and 0.30 mM octanoic acid datasets

**Additional file 8: Table S8.** List of tolerance determinants that are shared between 75 mM acetic acid and 14 mM butyric acid datasets.

**Additional file 9: Table S9.** List of tolerance determinants that are shared between 75 mM acetic acid and 0.30 mM octanoic acid datasets.

**Additional file 10:Table S10.** List of tolerance determinants that are shared between 14 mM butyric acid and 0.30 mM octanoic acid datasets.

**Additional file 11: Table S11.** Genes involved in vacuolar and vesicular function and transport. Table elaborated as described in Table 1.

**Additional file 12: Table S12.** Genes involved in macroautophagy. Table elaborated as described in Table 1.

**Additional file 13: Table S13.** Genes involved in transcription/ transcription regulation. Table elaborated as described in Table 1.

**Additional file 14: Table S14.** Genes involved in cytoplasmic translation. Table elaborated as described in Table 1.

**Additional file 15: Table S15.** Genes involved in mitochondrial translation. Table elaborated as described in Table 1.

**Additional file 16: Figure S1.** Comparison of growth by spot assays of the *Saccharomyces cerevisiae* BY4741 cell suspensions plated in solid YPD medium supplemented or not with acetic acid (70, 80, 90 and 100 mM), butyric acid (10, 12, 15, and 20 mM), and octanoic acid (0.22, 0.25 and 0.30 mM), at pH 4.5. Yeast cell suspensions used as inocula for spot assays were prepared using cells harvested in the exponential phase of growth (culture OD_600nm_ = 0.5 ± 0.05). Cell suspensions were diluted in sterile water to an OD_600nm_ = 0.25 ± 0.005 (**a**) and this solution was used to prepare 1:5 (**b**), 1:25 (**c**), 1:125 (**d**), and 1:625 (**e**) diluted suspensions. Susceptibility phenotypes were registered after 48 h of incubation at 30 ℃.

**Additional file 17: Figure S2.** Visual description of the criteria used to define the different levels of susceptibility to acetic (C2), butyric (C4) and octanoic (C8) acids of the deletion mutant strains tested. Wild-type and deletion mutant strains were spotted onto solid YPD medium (pH 4.5) supplemented with 75 mM C2, 14 mM C4 or 0.30 mM C8. Two levels of susceptibility were defined: (+) when the growth inhibition of the single mutant strains was minor to moderate or (++) referring to total growth inhibition compared to wild-type. “0” corresponds to an absence of a detectable susceptibility phenotype.

## Data Availability

The authors confirm that all of this study data is available within the article and its supplementary information.
