# Correction: Shared and more specifc genetic determinants and pathways underlying yeast tolerance to acetic, butyric, and octanoic acids

**DOI:** 10.1186/s12934-024-02429-7

**Published:** 2024-06-14

**Authors:** Marta N. Mota, Madalena Matos, Nada Bahri, Isabel Sá-Correia

**Affiliations:** 1 iBB—Institute for Bioengineering and Biosciences, Instituto Superior Técnico, Universidade de Lisboa, Av. Rovisco Pais, 1, Lisbon, 1049-001 Portugal; 2 Department of Bioengineering, Instituto Superior T?cnico, Universidadede Lisboa, Av. Rovisco Pais, 1, Lisbon, 1049-001 Portugal; 3 i4HB—Institute for Health and Bioeconomy, Instituto Superior Técnico, Universidade de Lisboa, Av. Rovisco Pais, 1, Lisbon, 1049-001 Portugal

**Correction: Microbial Cell Factories (2024) 23 :71**.

10.1186/s12934-024-02309-0

Following publication of the original article [1], the authors identified an error in Table 1.

“Gene/ORF Description of the encoded protein function Susceptibility C2 C4 C8” was mistakenly placed in middle of the Table 1. The correct Table 1 is given below.,.

The link of Table S2 should be redirected to Figure S2.

In addition to the genes shared by the three datasets, the butyric acid and octanoic acid datasets shared the highest number of identified genes in common [71] compared with any other combination of two acids. - it is supposed to be (71) and not linked to the reference 71.

In the legend of Table 1: The part to be removed " Table elaborated as described in Table 1” and the heading between VPS24 and VPS25 lines.

In the legend of Table 2: “The table includes the list of genes, involved in vacuolar acidification, identified in this study as determinants of yeast tolerance to acetic acid (C2), butyric acid (C4), or octanoic acid (C8). Table elaborated as described in Table 1.” to be added.

The remaining tables of the legend “Table elaborated as described in Table 2” should be replaced with “Table elaborated as described in Table 1”.

