# Supplementary figures and images for: Shared and more specific genetic determinants and pathways underlying yeast tolerance to acetic, butyric, and octanoic acids
Source: Microb Cell Fact. 2024 Feb 29;23:71. doi: 10.1186/s12934-024-02309-0 (PMC10903034; doi:10.1186/s12934-024-02309-0)

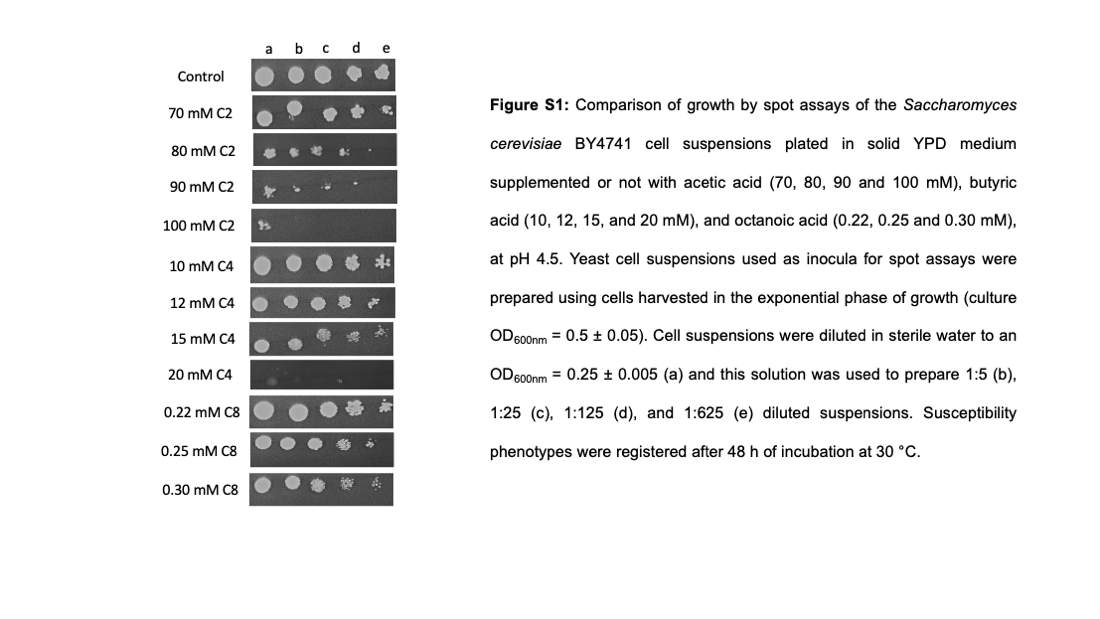

Supplement: Supplementary file 16 — Additional file 16: Figure S1. Comparison of growth by spot assays of the Saccharomyces cerevisiae BY4741 cell suspensions plated in solid YPD medium supplemented or not with acetic acid (70, 80, 90 and 100 mM), butyric acid (10, 12, 15, and 20 mM), and octanoic acid (0.22, 0.25 and 0.30 mM), at pH 4.5. Yeast cell suspensions used as inocula for spot assays were prepared using cells harvested in the exponential phase of growth (culture OD600nm = 0.5 ± 0.05). Cell suspensions were diluted in sterile water to an OD600nm = 0.25 ± 0.005 (a) and this solution was used to prepare 1:5 (b), 1:25 (c), 1:125 (d), and 1:625 (e) diluted suspensions. Susceptibility phenotypes were registered after 48 h of incubation at 30 ℃. [file 12934_2024_2309_MOESM16_ESM.tiff]

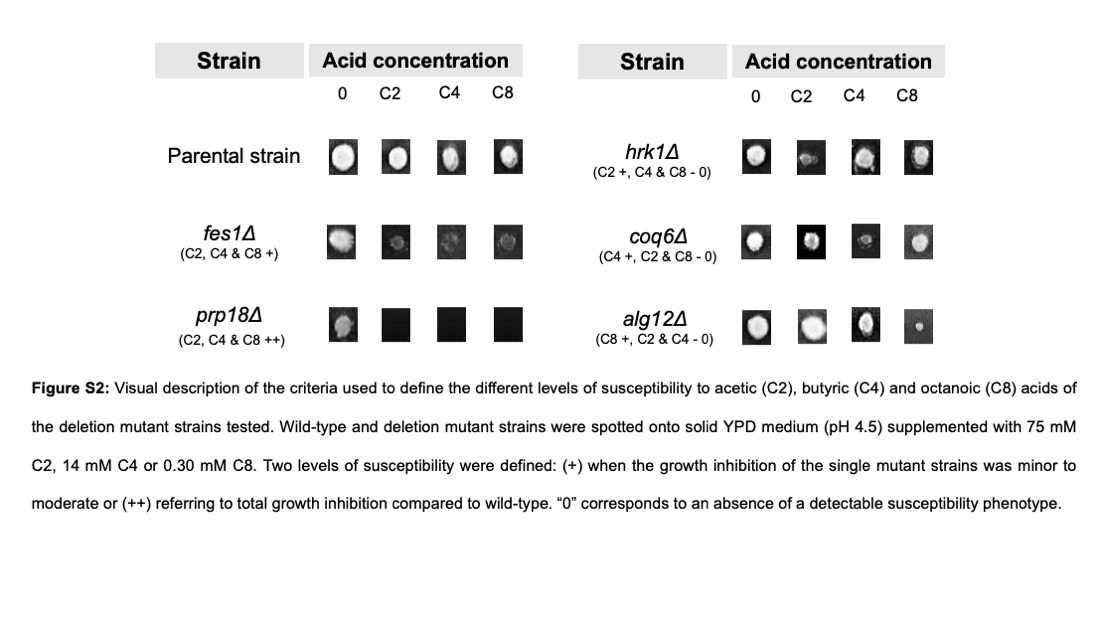

Supplement: Supplementary file 17 — Additional file 17: Figure S2. Visual description of the criteria used to define the different levels of susceptibility to acetic (C2), butyric (C4) and octanoic (C8) acids of the deletion mutant strains tested. Wild-type and deletion mutant strains were spotted onto solid YPD medium (pH 4.5) supplemented with 75 mM C2, 14 mM C4 or 0.30 mM C8. Two levels of susceptibility were defined: (+) when the growth inhibition of the single mutant strains was minor to moderate or (++) referring to total growth inhibition compared to wild-type. “0” corresponds to an absence of a detectable susceptibility phenotype. [file 12934_2024_2309_MOESM17_ESM.tiff]
